# Stress guides in generic static mechanical metamaterials

**DOI:** 10.1093/nsr/nwae110

**Published:** 2024-03-22

**Authors:** Aoxi Wang, Chang Qing Chen

**Affiliations:** Department of Engineering Mechanics, Center for Nano and Micromechanics and Key Laboratory of Applied Mechanics, Tsinghua University, Beijing 100084, China; Department of Engineering Mechanics, Center for Nano and Micromechanics and Key Laboratory of Applied Mechanics, Tsinghua University, Beijing 100084, China

**Keywords:** stress guides, space-time duality, mechanical metamaterials, non-Hermitian

## Abstract

The confinement of waves within a waveguide can enable directional transmission of signals, which has found wide applications in communication, imaging, and signal isolation. Extending this concept to static systems, where material deformation is piled up along a spatial trajectory, remains elusive due to the sensitivity of localized deformation to structural defects and impurities. Here, we propose a general framework to characterize localized static deformation responses in two-dimensional generic static mechanical metamaterials, by exploiting the duality between space in static systems and time in one-dimensional non-reciprocal wave systems. An internal time-reverse symmetry is developed by the space-time duality. Upon breaking this symmetry, quasi-static load-induced deformation can be guided to travel along a designated path, thereby realizing a stress guide. A combination of time-reverse and inversion symmetries discloses the parity-time symmetry inherent in static systems, which can be leveraged to achieve directional deformation shielding. The tailorable stress guides can find applications in various scenarios, ranging from stress shielding and energy harvesting in structural tasks to information processing in mechanical computing devices.

## Teaser

Teaser Tailoring static stress guides, similar to wave guides in dynamic systems, becomes possible in mechanical metamaterials.

## INTRODUCTION

Unlike bulk waves, a guided wave can propagate in a restricted area over a long distance with its domain boundaries serving as a waveguide. This includes electronic waves in a wire [1], electromagnetic waves in an optical fiber [2], acoustic waves in a pipe [3], and coupled elastic waves in beams [4] and plates [5]. As energy is confined in the waveguide and free from coupling with the surroundings during propagation, guided waves show high-quality transmission with low energy dissipation [6–8] and have been extensively exploited in modern communication technology, imaging, and structural health monitoring [9–11]. Extending this concept to static systems, wherein load-induced stress is directionally guided, is also appealing due to potential applications in stress cloaking [12], energy harvesting [13], and information processing [14,15]. Efforts have been made to trap and guide the localized static deformation in mechanical metamaterials, including the defect-induced patterned static deformations in origami [16] and kirigami [17], unidirectional stress focusing [18,19] and selective buckling [20] in topological Maxwell lattices, phase transformation in cellular materials [21], and temperature-sensitive deformations in thermally actuated composite metamaterials [22]. However, compared to dynamic guided waves, the directional navigation of stress guides in the form of load-induced localized static deformation of generic mechanical metamaterials is more challenging. One reason is that localized static deformations are usually sensitive to disorders and defects since the absence of an inertial effect deprives static deformation of the dynamic stability to detour them [19,23]. More importantly, the absence of theories that faithfully predict the correlation between localized static deformation and the microstructure of generic mechanical metamaterials, such as the dispersion relationship in wave dynamics [24], prevents the realization of tailorable stress guides.

Here, we tackle these challenges and construct a general framework for quantifying the localized static deformation response in generic mechanical metamaterials, provided that the periodicity of their microstructures is retained. We unveil the duality between space in two-dimensional (2D) generic static mechanical metamaterials and time in one-dimensional (1D) wave systems, which enables us to map the 2D localized deformation field to 1D non-reciprocal wave motion. This mapping allows us to characterize the deformation using the non-Hermitian band theory, which in turn facilitates the construction of directional stress guides. We recast internal symmetries, including the time-reverse symmetry (TRS) and the parity-time symmetry (PTS) [25], to static systems and show their capability for guiding directional stress. Within this framework, we also disclose a reciprocal topological zero mode (TZM) supported by intrinsic PTS, allowing for selective deformation. We further validate the generality of our theory by applying it to non-locally coupled lattices that go beyond the Maxwell limit, non-rectangular lattices with non-orthogonal basis vectors, and continuous elastic systems.

## RESULTS

### Space-time dualities and symmetries in X-braced lattices

**Figure 1.**
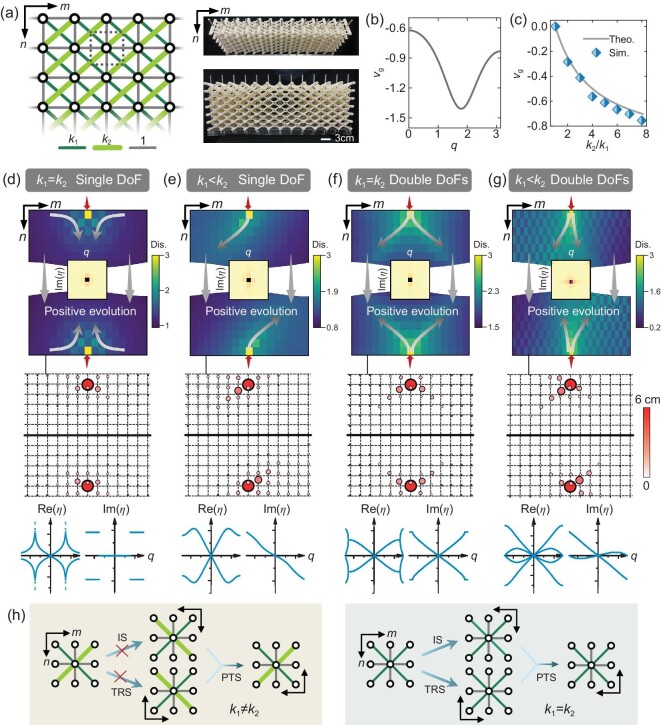
Signal transmission and symmetries in the static Rayleigh model. (a) The left panel is a schematic of the X-braced lattice. The stiffnesses of the horizontal and vertical bars are unitized and those of the diagonal bars are ${{k}_1}$ and ${{k}_2}$, respectively. The dashed box marks the unit cell. The right panel is a top and a front view of the 3D-printed lattice. (b) Group velocity of (e) in the half BZ. (c) Simulated group velocity of a Gaussian wave packet with various stiffness contrasts and a single DoF. (d–g) The top panels are simulated displacement fields subjected to a point load (red arrows) applied on the top or bottom boundary, with the reciprocal fields shown in the insets. The middle panels are the corresponding experimental measurements. The lower panels show the real and imaginary parts of the decay spectra, where the imaginary bands are doubly degenerate. (h) Illustrations of IS, TRS, and PTS from the lattice reflection symmetries along the *n* and *m* axes. The stiffnesses are ${{k}_1} = {{k}_2} = 6$ for (d) and (f), ${{k}_1} = 1$ and ${{k}_2} = 6$ for (b) and (e), ${{k}_1} = 0.3$ and ${{k}_2} = 6$ for (g), respectively.

A paradigm of the considered mechanical metamaterial is a planar X-braced lattice with the normalized stiffness between adjacent nodes being ${{k}_1}$, ${{k}_2}$, and 1, respectively, see the left panel of Fig. 1a. The lattice constant is set as 1 with an arbitrary unit. The right photographs are of the 3D-printed monolithic lattice (Supplementary Section XIII). For mathematical simplicity, the nodes are restricted to translate vertically in the current stage, while our theory is universal and is accessible to lattices with two degrees of freedom (DoFs) as well (see below). Applying a point load at the center of either the top or bottom boundary, the simulated and experimentally measured displacement fields for symmetric (${{k}_1} = {{k}_2}$) and asymmetric (${{k}_1} \ne {{k}_2}$) lattices are shown in the top and middle panels of Fig. 1d and e, respectively. The deformation for ${{k}_1} = {{k}_2}$ is seen to be symmetrically distributed and is highly localized in the middle bulk region (Fig. 1d), while for ${{k}_1} \ne {{k}_2}$, the deformation is guided towards a diagonal path, see the curved arrows in Fig. 1e. These deformation modes, despite being evident when considering the symmetries of the lattice geometries, can essentially be interpreted by the transmission theory for non-reciprocal wave motions and serve as the building blocks for constructing the directional stress guides, as shown in the following.

We focus on the case of a point load applied on the top boundary. Generally, an irregular nodal displacement at $n = 0$ induced by a point load can be decomposed into a series of sinusoidal patterns via the Fourier transform. As a result, the deformation response inside the lattice ($n > 0$) can be considered as a superposition of the sinusoidal patterns due to linearity. Every sinusoidal load drives a static Rayleigh (SR) displacement field [26,27] with an exponential decay rate away from the boundary, i.e. ${{u}_{m,n}} = {{e}^{ - \eta n}}{{e}^{iqm}}$ with *q* the wavenumber and $\eta ( q )$ the decay factor. The real and imaginary parts of $\eta $ characterize the decay rate and phase accumulation of the boundary load during its spatial evolution along the *n* axis. This displacement field resembles a wave solution but with the dimensional enhancement from $1 \oplus 1$D space-time in dynamics to 2D spaces in statics, along with the conversion from time-harmonic to space-attenuated due to Saint-Venant's principle [28]. To exemplify the argument, we consider a semi-infinite plane-strain continuum with isotropic elasticity. The horizontal/vertical displacement fields are ${{u}_{m/n}}( {m,n} )$. Assuming ${{u}_m}( {m,n} ) = C{{u}_n}( {m,n} )$ with a complex coefficient *C* and substituting it into the governing Lame's equation yield (Supplementary Section III)


(1) \begin{eqnarray*}
&&\displaystyle\frac{{2{{C}^2}\left( {1 - \nu } \right) - \left( {1 - 2\nu } \right)}}{{{{C}^2}\left( {1 - 2\nu } \right) - 2\left( {1 - \nu } \right)}}\frac{{{{\partial }^2}{{u}_{n}}\left( {m,n} \right)}}{{\partial {{m}^2}}}\\
&&\quad \quad +\, \displaystyle\frac{{{{\partial }^2}{{u}_n}\left( {m,n} \right)}}{{\partial {{n}^2}}} = 0,
\end{eqnarray*}

where $\nu $ is Poisson's ratio. Eq. (1) is mathematically equivalent to the 1D D'Alembert equation for transverse wave motion, ${{c}^2}\partial _m^2{{u}_n}( {m,t} ) - \partial _t^2{{u}_n}( {m,t} ) = 0$ with *c* the sound velocity, but with a substitution of time *t* in dynamics to space *n* in statics. Additionally, the minus sign in the hyperbolic D'Alembert equation is converted to a plus sign in Eq. (1), which is elliptic. The change implies that the imaginary phase factor $i\omega $ should be replaced by a real decay factor $\eta $, thereby yielding the SR solution. When plugged into Eq. (1), this solution gives rise to linear dispersion $\eta ( q ) = \pm q$ along $C = - i$ and *i*. Therefore, Eq. (1) can be simplified as a 2D Laplace equation, $\partial _m^2{{u}_n} + \partial _n^2{{u}_n} = 0$, which is consistent with the isotropic character of the continuum. The Laplace equation can be reformulated in the Schrödinger-form, $i\eta {{\partial }_t}{{u}_n} = - \partial _m^2{{u}_n}$ by adopting the ansatz ${{u}_n}( {m,t} ) = {{e}^{ - i\eta t}}{{u}_n}( {m,0} )$ and an imaginary gauge transform [27,29], $n = ti$. In practice, the implementation of the continuous boundary load can be nontrivial, but in Supplementary Section IV we show that discretizing Eq. (1) yields a lattice equilibrium equation that asymptotically converges to a symmetric X-braced lattice when ${{k}_1} \to 0$. The pin-joint structure of this lattice provides a practically accessible route for applying the delicate boundary condition. The above analysis can be readily extended to 3D continua and associated lattice materials, as elucidated in Supplementary Section IX.

Based on the mathematical equivalence of both the displacement fields and governing equations, the static deformation response in 2D lattice materials can be characterized by the band theory and symmetry identification for 1D wave motion. First, the bulk equilibrium equation of the X-braced lattice can be formulated as the following eigen-equation about the effective Bloch Hamiltonian, ${{H}_{{\mathrm{SR}}}}( q )$ [28]


(2) \begin{eqnarray*}
&&{{H}_{{\mathrm{SR}}}}\left( q \right)\Big\{ \begin{array}{@{}c@{}} 1\\ \lambda \left( q \right) \end{array} \Big\}\\
&&= \left[ {\begin{array}{@{}*{2}{c}@{}} 0&1\\
{ - \displaystyle\frac{{1 + {{k}_1}{{e}^{ - iq}} + {{k}_2}{{e}^{iq}}}}{{1 + {{k}_1}{{e}^{iq}} + {{k}_2}{{e}^{ - iq}}}}}&{\displaystyle\frac{{2\left( {2 + {{k}_1} + {{k}_2} - \cos \left( q \right)} \right)}}{{1 + {{k}_1}{{e}^{iq}} + {{k}_2}{{e}^{ - iq}}}}} \end{array}} \right]\\
&&\times \,\Big\{ \begin{array}{@{}c@{}} 1\\ \lambda \left( q \right) \end{array} \Big\}\, {\mathrm{ = }}\, \lambda \left( q \right)\Big\{ \begin{array}{@{}c@{}} 1\\ \lambda \left( q \right) \end{array} \Big\},
\end{eqnarray*}

where the eigenvalue $\lambda ( q ) = {{e}^{ - \eta ( q )}}$. The dispersion about $\lambda - q$ or $\eta - q$, dubbed as the decay spectrum [28], can be solved from Eq. (2). The spectrum is real when ${{k}_1} = {{k}_2}$ due to the quasi-Hermiticity [30] of ${{H}_{{\mathrm{SR}}}}( q )$, but it becomes complex once ${{k}_1} \ne {{k}_2}$. The nonzero imaginary part signifies a nonvanishing group velocity of the wave packet. In our static model, the wave packet is mapped to a compact deformation pattern centered at a site/wavenumber in the real/reciprocal space and propagates along the *m* axis during *n*-directional spatial evolution [26,27,31]. The peak response of the wave packet is determined by $\Delta {{m}_{\max }} = ( {{{d{\mathop{\mathrm{Im}}\nolimits} ( \eta )} \mathord{/ {\vphantom {{d{\mathop{\mathrm{Im}}\nolimits} ( \eta )} {dq}}}} {dq}}} )\Delta n$ (Supplementary Section V). The transmission rate of the peak characterizes the skewing velocity of the clustered deformation mode away from its injected location and is termed as the group velocity, i.e. ${{v}_g}( q ) = {{\Delta {{m}_{\max }}} \mathord{/ {\vphantom {{\Delta {{m}_{\max }}} {\Delta n}}}} {\Delta n}} = {{d{\mathop{\mathrm{Im}}\nolimits} ( \eta )} \mathord{/ {\vphantom {{d{\mathop{\mathrm{Im}}\nolimits} ( \eta )} {dq}}}} {dq}}$. This is a direct generalization from wave dynamics [24] as ${\mathop{\mathrm{Im}}\nolimits} ( \eta )$ encodes the phase information. Therefore, it is possible to concentrate the stress field on a given diagonal path by imposing a nonzero group velocity of the injected wave packet.

For a sinusoidal load applied on the top boundary, it evolves along the $+ n$ axis with a positive decay rate, ${\mathop{\mathrm{Re}}\nolimits} ( \eta ) > 0$. A ‘time-reversal’ flips the *n* axis and the subsequently reversed state is $u_{m,n}^T = {{e}^{\eta n}}{{e}^{iqm}}$, which is an amplified state with a growing intensity along the $+ n$ axis and satisfies the basic requirements of a time-reversed pulse [32], $u_{m, - n}^T = {{u}_{m,n}}$ and $v_{m, - n}^T = - {{v}_{m,n}}$ with ${{v}_{m,n}} = {{d{{u}_{m,n}}} \mathord{/ {\vphantom {{d{{u}_{m,n}}} {dn}}}} {dn}} = - \eta {{u}_{m,n}}$ the velocity-emulated displacement gradient. In fact, the time-reversed state corresponds to the SR mode with an external load applied on the bottom boundary and decays along the $- n$ axis, which in turn mimics a growing state when tracking the positive direction of time flow. A static system has TRS only if $u_{m,n}^T$ satisfies the equilibrium equation. By contrast, the space-inversion is taken as the reflection along the *m* axis that serves as a real physical space, i.e. $u_{m,n}^P = {{u}_{ - m,n}} = {{e}^{ - \eta n}}{{e}^{ - iqm}}$. A symmetric X-braced lattice (or an isotropic continuum) sustains both the TRS and inversion symmetry (IS), as can be shown by substituting $u_{m,n}^T$ and $u_{m,n}^P$ into Eq. (2) (or Eq. (1)). Intuitively, the preserved TRS and IS are attributed to the independent reflection symmetries of the symmetric lattice (the continuum) along the *n* and *m* axes, respectively. Here, the concretization of time enables the observation of internal TRS via crystalline symmetry. On the other hand, the lattice symmetries, either spatial or nonspatial, can be extracted from the Hamiltonian. TRS (IS) tailors a unitary symmetry *T* (*P*) such that $T{{H}_{{\mathrm{SR}}}}( q ){{T}^{ - 1}} = H_{{\mathrm{SR}}}^{ - 1}( q )$ ($P{{H}_{{\mathrm{SR}}}}( q ){{P}^{ - 1}} = {{H}_{{\mathrm{SR}}}}( { - q} )$). The involvement of the inverse of the effective Hamiltonian in the redefined TRS is to guarantee that $q( \eta ) = q( { - \eta } )$, since the eigenvalue is not the ‘effective energy’ $\eta $ itself. A discussion about the unitarity of the TRS operator is provided in Supplementary Section II. For symmetric lattices, we have $T = {{\sigma }_x}$ the Pauli matrix and $P = I$ the identity matrix (the identification of symmetry operators is given in Supplementary Section VI). By contrast, both TRS and IS are collapsed in asymmetric lattices owing to the broken reflection symmetries along the two axes.

Having recast the basic symmetries and redefined the group velocity, the static deformations in Fig. 1d and e can be reinterpreted from a ‘dynamic’ perspective. The displacement at $n = 0$ excited by a point load resembles a Gaussian wave packet [33] with a center wavenumber at $q = 0$, see the insets of Fig. 1d and e for the 2D Fourier transform of the deformation fields, which lie in the reciprocal space spanned by *q* and ${\mathop{\mathrm{Im}}\nolimits} ( \eta )$. Consequently, for symmetric lattices with a real decay spectrum (${{v}_g} = 0$), the wave packet does not propagate and is localized at its initial position (middle column region), forming a standing wave. For asymmetric lattices, on the contrary, TRS is broken so that ${{v}_g}$ is negative in the whole Brillouin zone (BZ) for both the original (${\mathop{\mathrm{Re}}\nolimits} ( \eta ) > 0$) and time-reversed (${\mathop{\mathrm{Re}}\nolimits} ( \eta ) < 0$) states (Fig. 1b), inducing a chiral transport of the wave packet with its center unidirectionally moving towards the left boundary. This directional transmission facilitates the confinement of the stress field in a spatial trajectory, and such a process, when mapped back to the dynamic system, corresponds to a unidirectional propagation of the wave packet in a TRS broken medium. Note that the sign of the group velocity, which dictates the skewing orientation of the wave packet during its spatial evolution, is defined on a global basis with the positive evolution direction being downward. The simulated group velocity for different stiffness contrasts (Fig. 1c) further verifies our analysis (Supplementary Section XII). The magnitude of the group velocity increases monotonically with the degree of non-reciprocity (${{{{k}_2}} \mathord{/ {\vphantom {{{{k}_2}} {{{k}_1}}}}} {{{k}_1}}}$), as expected. The simulation result is slightly larger than the theoretical one, since the redundant components of the wavenumber accelerate the wave packet ($| {{{v}_g}} |$ takes minimum at $q = 0$, see Fig. 1b). Nevertheless, $| {{{v}_g}( 0 )} |$ is strictly restricted within the upper limit 1. When ${{k}_2}$>>${{k}_1}$ ∼ *O*(1), the decay spectrum is simply $\eta = - iq$ and we have ${{v}_g} = - 1$, emulating a nondispersive medium. Furthermore, the peak response of a point load ${{F}_{m,0}} = {{F}_0}{{\delta }_{pm}}$ with $\delta $ being the Kronecker symbol and integer *p*, is constrained at the sites $( {{{m}_{\max }} = p - n,n} )$, which naturally yields that ${{v}_g} = {{\Delta {{m}_{\max }}} \mathord{/ {\vphantom {{\Delta {{m}_{\max }}} {\Delta n}}}} {\Delta n}} = - 1$.

Two remarks should be made about this model. First, for symmetric lattices, if we release the horizontal DoF, we have ${\mathop{\mathrm{Im}}\nolimits} ( \eta ) \ne 0$ even with ${{k}_1} = {{k}_2}$, suggesting a nonzero group velocity. Nevertheless, because in this case ${\mathop{\mathrm{Im}}\nolimits} ( \eta )$ contains two nondegenerate bands that are symmetric about the BZ center (bottom of Fig. 1f) for preserved TRS, the wave packet travels symmetrically towards the left and right boundaries with an equal skewing rate (top and middle of Fig. 1f) and forms a bidirectional stress guide. For asymmetric lattices with released DoFs, the two branches of ${\mathop{\mathrm{Im}}\nolimits} ( \eta )$ have opposite signs and magnitudes of their slopes (bottom of Fig. 1g), for which the incident wave packet moves towards the left and right boundaries with unequal traveling angles, as visualized in the top and middle panels of Fig. 1g for the asymmetric bifurcation of the bulk displacement fields (see details in Supplementary Section VI). Second, despite TRS and IS being simultaneously broken in asymmetric lattices, the combination of the two, namely, $u_{m,n}^{PT} = {{u}_{ - m, - n}} = {{e}^{\eta n}}{{e}^{ - iqm}}$, is still an admissible state of Eq. (2), as confirmed by $PT{{H}_{{\mathrm{SR}}}}( q ){{( {PT} )}^{ - 1}} = H_{{\mathrm{SR}}}^{ - 1}( { - q} )$ or $\eta ( { - q} ) = - \eta ( q )$. This unitary symmetry can be termed as the combined PTS for the joint invariance under time-reverse and space-inversion, even if either of the constituent symmetries is broken [25]. From a geometrical point of view, the intrinsic PTS is related to the 2D inversion symmetry of the X-braced lattice within the $m - n$ plane, as illustrated in Fig. 1h. Interestingly, the PTS has a similar effect on the decay spectrum compared to the particle-hole symmetry (PHS) on the phonon spectrum, and the latter is also intrinsic for mechanical wave systems even when the TRS is broken [30,34,35].

A profound analogy between the clean Hatano-Nelson (HN) model in quantum mechanics [36] and our SR model can be identified. A simple realization of the HN model is a monoatomic mass-spring chain with asymmetric coupling stiffnesses [37]. The TRS is sustained in the HN model, since the originally amplified forward state becomes an attenuated backward state under time reversal [38], which still follows Newton's law. In this sense, the HN model only favors ‘unidirectional amplification’ but not ‘unidirectional transmission’, and the latter is unique to our SR model with broken TRS and IS. A deeper symmetry analysis uncovering the correlation between the two models is given in Supplementary Section II. In the long wavelength limit, the continuous governing equation for the HN model is ${{c}^2}\partial _m^2{{u}_m} - \partial _t^2{{u}_m} - {{2\varepsilon {{c}^2}{{\partial }_m}{{u}_m}} \mathord{/ {\vphantom {{2\varepsilon {{c}^2}{{\partial }_m}{{u}_m}} h}}} h} = 0$ with $\varepsilon $ representing the degree of non-reciprocity [37], and is $( {1 + {{k}_1} + {{k}_2}} )( {\partial _m^2{{u}_n} + \partial _n^2{{u}_n}} ) + 2( {{{k}_1} - {{k}_2}} ){{\partial }_m}{{\partial }_n}{{u}_n} = 0$ for the SR model, respectively. A nonzero $\varepsilon $ breaks IS while preserving TRS in the HN model, and this equation is mathematically equivalent to the Klein-Gordon equation under an imaginary gauge field (Supplementary Section I). For the SR model, the continuous equation degenerates to isotropic media when ${{k}_1} = {{k}_2}$, whereas an odd differential term that mixes the space (*m*) and time (*n*) is attained when ${{k}_1} \ne {{k}_2}$, for which the TRS and IS are simultaneously broken and combined PTS is preserved. Hence, we cannot break the symmetry along one of the two dimensions while preserving the other, for which the PTS is called ‘intrinsic’. Conversely, the space-time is decoupled in the HN model and the broken reciprocity in the space domain does not violate TRS.

### Programming localized static deformations in heterostructures

**Figure 2.**
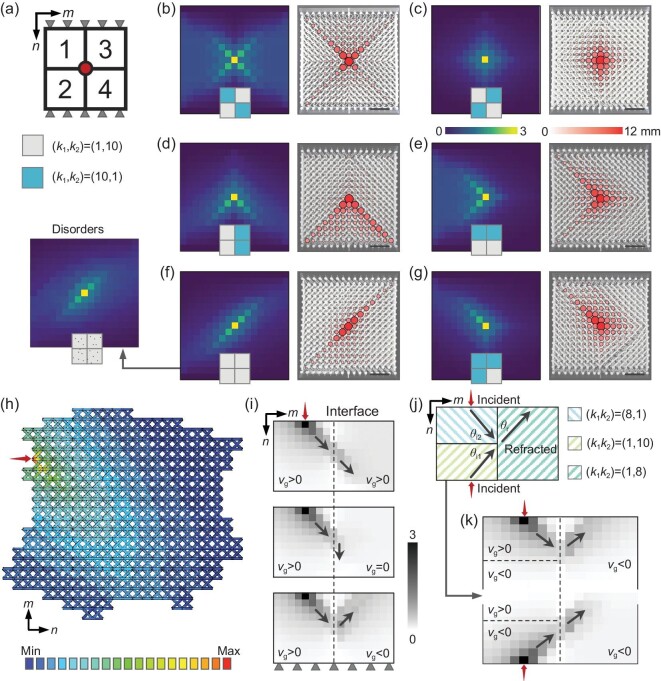
Programmed static deformation fields in heterostructures. (a) Schematic of a heterostructure composed of four X-braced lattices with a point load applied on the junction (red dot). (b–g) The left panels show simulated displacement fields of the heterostructures with various permutations of the constituent parts. The right panels are the corresponding experimental measurements in the monolithic frame-like lattices (the scale bars are 6 cm). There are randomly distributed ground springs connected with the lattice sites in the inset of (f) to demonstrate the transmission robustness against disorders. (h) The simulated displacement field of an asymmetric X-braced lattice with irregular boundary and a point load. The stiffnesses are $( {{{k}_1},{{k}_2}} ) = ( {1,10} )$. (i) Simulated displacement fields of the heterostructures composed of two X-braced lattices, with a point load applied on the top boundary of the left part. The stiffnesses of the left part are $( {{{k}_1},{{k}_2}} ) = ( {8,1} )$, while they are $( {{{k}_1},{{k}_2}} ) = ( {4,1} )$ (top panel), $( {1,1} )$ (middle panel), and $( {1,8} )$ (bottom panel) for the right part, respectively. (j) Schematic of a heterostructure composed of three X-braced lattices. (k) Simulated displacement fields of (j).

Compared to directional wave motion, the confinement and guide of a local static deformation is more difficult due to the lack of inertial effects that push it to travel [28,39,40]. Our theory thus provides a general framework for the manipulation of static deformation response via band structure analysis and symmetry identification of the host lattice. Consider a heterostructure assembled from four X-braced lattices with two stiffness patterns, see Fig. 2a. A point load applied at the junction of the four parts excites a pair of wave packets attenuating along the $+ n$ and $- n$ directions and corresponding to the original state and time-reversed state, respectively. Depending on the symmetry, the static deformation in each part can be guided along a designated path. Then, the collective deformation of the heterostructure can be patterned via different permutations of the constituent parts, giving rise to a tailorable stress guide. As a proof of concept, Fig. 2b**–**g reports the simulated (left panels) and measured (right panels) displacement fields in six different configurations, see Supplementary Sections XII and XIII for details of the simulations and experiments. The diverse deformation patterns excited by the point load can be well predicted by the aforementioned symmetry analysis. For example, in Fig. 2b, the wave packet propagates unidirectionally towards the left and right boundaries in part-2 and part-4 owing to their reversed group velocities, while the time-reversed state propagates in part-1 and part-3 with the same group velocity pertaining to part-4 and part-2 thanks to the intrinsic PTS protection. By contrast, the injected wave packet in Fig. 2c tends to propagate rightward (leftward) in part-2 and part-3 (part-1 and part-4) and is not permitted under the current configuration, inducing deformation trapping at the vertical interface that is free from permeating deeply into the bulk. This serves as a mechanical analog to non-reciprocal light funneling in photonic crystals, where the injected light beam is trapped by the domain wall due to the skin effect [41]. Transmission robustness is verified by adding randomly distributed ground springs to the lattice sites in Fig. 2f, see the inset, where the excited deformation is still guided towards the diagonal path. It is also meaningful to investigate the interaction of directional stress focusing, fracture toughness, and network topology using the method proposed in this work and fracture mechanics [42], even though it is beyond the scope of our study. Besides, the transmission is also irrelevant to the lattice geometry, as confirmed in Fig. 2h for an asymmetric X-braced lattice with irregular boundaries, where unidirectional stress focusing is retained.

Furthermore, a combination of two X-braced lattices with opposite chirality can induce a deflection of the propagating wave packet when encountering the domain wall, owing to the abrupt change in the group velocity, see Fig. 2i. The stiffnesses of the left part are fixed as $( {{{k}_1},{{k}_2}} ) = ( {8,1} )$, so that a wave packet triggered by a set of point loads (red arrows) can propagate towards the interface. Three sets of stiffnesses are selected for the right part, i.e. $( {{{k}_1},{{k}_2}} ) = ( {4,1} )$, $( {1,1} )$, and $( {1,8} )$. The wave packet passes through the interface in the top panel of Fig. 2i with negligible deflection owing to the fact that the two parts have almost the same group velocity, while it is arrested by the interface in the middle panel of Fig. 2i since the right part does not support any traveling channel (${{v}_g} = 0$). Especially, negative refraction is found in the bottom panel of Fig. 2i due to the revered group velocity of the two parts, reminiscent of the chirality-reverse induced negative refraction in a time-modulated Floquet lattice [43]. On this basis, we can also achieve an asymmetric negative refraction effect where the reversal of incident angles induces a transition from positive refraction to negative refraction [44]. This is fulfilled in a heterostructure composed of three subparts with their stiffnesses marked in Fig. 2j. Here, the wave packet incident from the lower (upper) left part has a negative (positive) group velocity and an incident angle ${{\theta }_{i1}}$ (${{\theta }_{i2}}$), and we have ${\mathop{\mathrm{sgn}}} ( {{{\theta }_{i1}}} ) = - {\mathop{\mathrm{sgn}}} ( {{{\theta }_{i2}}} )$. Then, the wave packets collide with the vertical interface and deflect their traveling angles to a common value ${{\theta }_r}$ when refracted into the right part, see the simulated displacement fields in Fig. 2k. Hence, the wave packet incident from the lower left experiences positive refraction whereas the one incident from the upper left is negatively refracted upon approaching the interface, corroborating the asymmetric negative refraction effect in our static systems.

**Figure 3.**
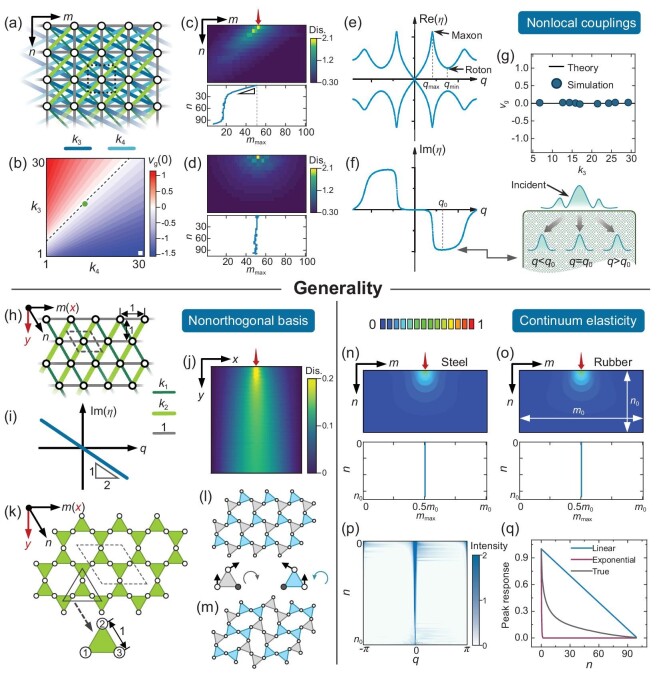
Generality of the transmission theory for static deformation fields. (a) Schematic of a modified X-braced lattice with next-nearest-neighbor couplings, ${{k}_3}$ and ${{k}_4}$. (b) Colormap of ${{v}_g}( 0 )$ in the ${{k}_3} - {{k}_4}$ parameter space, where ${{k}_1} = 1$ and ${{k}_2} = 10$. (c and d) Top panels are simulated displacement fields under a point load applied on the top boundary. The bottom panels are the corresponding spatial evolutions of the wave packet peaks. (e and f) Real (e) and imaginary (f) parts of the decay spectrum. The inset of (f) gives an illustration of the beam splitting in nonlocal static lattices. (g) Simulated group velocities for parameters lying on the dashed black line of (b). The stiffnesses are $( {{{k}_3},{{k}_4}} ) = ( {1,30} )$ for (c), and $( {16.95,12.45} )$ for (d–f), respectively. Other stiffnesses are fixed as $( {{{k}_1},{{k}_2}} ) = ( {1,10} )$. (h) Schematic of a triangular lattice. (i) Corresponding imaginary decay spectrum for ${{k}_1} = {{k}_2}$. (j) Simulated displacement field under a point load applied on the top boundary. The displacement of every horizontal row is renormalized for better visualization. The stiffnesses are ${{k}_1} = {{k}_2} = 14.3$. (k) Schematic of a regular Kagome lattice. (l and m) Illustrations of two Guest-Huchinson modes, where the blue and gray triangles rotate counterclockwise and clockwise, respectively. (n and o) The top panels are simulated displacement fields for a steel (n) and a rubber (o) under a point load applied on the top boundary. The bottom panels are corresponding spatial evolutions of the wave packet peaks. (p) *m*-directional Fourier transforms of the normalized displacement fields in (o). (q) Estimated (blue and red lines) and true (black line) attenuations of the wave packet amplitudes in (o).

Note that previous studies have quantified the localized deformation responses in isostatic lattice materials at the verge of mechanical stability, e.g. triangular array [45] in 1D, square lattice [46] and kagome lattice [18–20] in 2D, and stacked kagome lattice [47] in 3D. In these studies, unidirectional stress focusing on the domain boundaries has been realized, by leveraging the zero-energy floppy deformation modes and the states of self-stress in a topologically nontrivial configuration. While these boundary modes are topologically protected and show robustness against structural imperfections and disorders, they are limited to a small group of lattices. In contrast, our theory is based on the band structure and is applicable to any periodic lattice materials. Additional exemplifications revealing the generality of our theory are shown in Fig. 3. Figure 3a shows a modified X-braced lattice with next-nearest-neighbor diagonal couplings, where the nonlocal effect drastically enriches the transmission properties of static lattice materials. Figure 3b shows the colormap of ${{v}_g}( 0 )$ in the ${{k}_3} - {{k}_4}$ parameter space. It can be found that the upper bound of $| {{{v}_g}( 0 )} |$ is improved compared to X-braced lattices, i.e. 2 for modified X-braced lattices and 1 for X-braced lattices. This improvement facilitates a larger traveling angle of the incident wave packet, as shown in Fig. 3c, corresponding to the parameters marked by the white square in Fig. 3b. Furthermore, there is a special set of parameters such that $| {{{v}_g}( 0 )} | = 0$, as marked by the dashed line in Fig. 3b, even if the nonlocal lattice is neither symmetric about the *m* nor *n* axes. As an illustration, we show in Fig. 3d the simulated displacement field and the spatial evolution of wave packet peaks for the parameters marked by the green dot in Fig. 3b, where a standing wave is observed. The associated decay spectra are shown in Fig. 3e and f. Note that the real decay spectrum displays an unusual roton-like dispersion [48], where there is a local maximum (referred to as ‘maxon’) and a minimum (referred to as ‘roton’) at ${{q}_{\max }}$ and ${{q}_{\min }}$, respectively. The imaginary spectrum exhibits a local minimum at ${{q}_0}$ and a negative (positive) group velocity for $q < {{q}_0}$ ($q > {{q}_0}$). This renders the nonlocal lattice a ‘beam splitting’ capability for separating the nonmonochromatic wave packet and facilitating the construction of multidirectional stress guides, as schematically shown in the inset of Fig. 3f. Figure 3g depicts the simulated group velocities for parameters lying on the dashed line of Fig. 3b, which are well constrained in the vicinity of $| {{{v}_g}( 0 )} | = 0$.

To further exemplify our theory in lattices with non-orthogonal basis vectors, a triangular lattice (Fig. 3h) is explored, where the group velocity for ${{k}_1} = {{k}_2}$ is calculated as ${{v}_g} = 0.5$ for arbitrary wavenumbers (Fig. 3i). After transforming the nominal group velocity to the orthogonal basis spanned by *x* and *y*, we obtain a standing wave solution, in compliance with the symmetric nature of a triangular lattice with ${{k}_1} = {{k}_2}$, see the simulated displacement field in Fig. 3j. Figure 3k shows a regular kagome lattice with a uniform linking stiffness and two DoFs per node, where the decay spectra are solved as ${{\eta }_1} = 0$ and ${{\eta }_2} = - iq$. These two modes are non-attenuated and correspond to the Guest-Hutchinson modes in isostatic lattices [49], as shown in Fig. 3l and m. The group velocities are ${{v}_{g,1}} = 0$ and ${{v}_{g,2}} = - 1$ and, when transformed to the orthogonal basis, are associated with the wave packets propagating towards the right and left boundaries upon applying a point load on the top boundary, which is consistent with the experimental observations in rubbers [50].

Our theory is also applicable to continuous elastostatics, provided that the decay spectrum can be well defined. For example, the decay spectrum of an isotropic continuum is $\eta ( q ) = \pm q$, giving rise to a zero group velocity and a standing wave solution of an applied point load. This result is immune to the constituent material properties, as demonstrated in Fig. 3n and o for steel (with Young's modulus 200 GPa and Poisson's ratio 0.26) and rubber (with Young's modulus 7.8 MPa and Poisson's ratio 0.47), which are extracted from the FEM software Abaqus (Supplementary Section XII). Besides, the real decay spectrum renders a qualitative estimation of the wave packet attenuation. Taking rubber as an example, Fig. 3p shows the *m*-directional Fourier transforms of the normalized displacement fields in every horizontal row. It can be seen that the wave packet is centered at $q = 0$ and $\pi $ in the vicinity of $n = 0$, whereas the short wave is evanescent and only $q = 0$ dominates in the deep bulk. Consequently, the attenuation of the wave packet amplitudes is sandwiched between the linear envelope (for $\eta = q = 0$) and exponential envelope (for $\eta = q = \pi $), as confirmed in Fig. 3q. More details are provided in Supplementary Sections VII and XII.

In fact, our study provides a general framework for identifying the bulk deformation response actuated by any paradigms of the external stimulus that can be decomposed as a series of SR modes and in any periodical lattice materials/continua where the decay spectrum can be well defined. We also note that there are no restrictions on the characteristic length scale of our method.

### Reciprocal topological zero modes in non-Hermitian rhombus lattices

**Figure 4.**
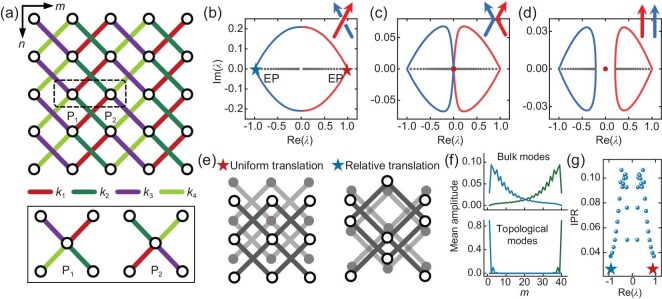
Non-Hermitian rhombus lattice. (a) Schematic of the compound rhombus lattice with asymmetric intra- and inter-cellular diagonal stiffnesses. The inset shows the two sublattices in a unit cell. (b–d) PBC (solid lines) and OBC (solid dots) spectra, with the TZMs marked by the red dots. The stiffnesses are $( {{{k}_1},{{k}_2},{{k}_3},{{k}_4}} ) = ( {3,2,1,4} )$ for (b), $( {1,2,1,4} )$ for (c), and $( {1,2,3,4} )$ for (d), respectively. The insets show the braid diagrams of the PBC bands. (e) Deformation modes of the two EPs: uniform translation of the entire lattice (left panel) and relative translation of two nested sublattices (right panel). (f) Mean on-site amplitudes for all bulk modes (top panel) and the paired TZMs (bottom panel) in (d). The green and blue lines correspond to the inner ($| \lambda | < 1$) and outer ($| \lambda | > 1$) bands, respectively, which form reciprocal pairs. (g) Inverse participation ratio (IPR) for the 38 bulk modes in (d). IPR approaches zero (one) when an eigenstate is extended (localized). The *m*-directional site number for an OBC system is $M = 40$ (20 unit cells).

In the previous sections, we present a general framework for identifying localized deformation fields in generic static mechanical metamaterials, by exploiting their band structures and symmetries. The inherent space-time duality facilitates the concretization of internal TRS and PTS in static systems, which can be employed to tailor edge modes in topologically nontrivial systems, as shown below. Consider a single DoF diatomic rhombus lattice shown in Fig. 4a. Using the SR solution, ${{{\boldsymbol{u}}}_{m,n}} = {{\lambda }^n}{{e}^{iqm}}{\boldsymbol{p}}$ with ${\boldsymbol{p}} = {{( {{{p}_1},{{p}_2}} )}^T}$ the sublattice polarization [26], the lattice equilibrium equation can be arranged as an eigen-equation, given by (Supplementary Section VIII)


(3) \begin{eqnarray*}
H\left( q \right)\left\{ \begin{array}{@{}l@{}} {\boldsymbol{p}}\\ \lambda {\boldsymbol{p}} \end{array} \right\} = \left[ {\begin{array}{@{}*{4}{c}@{}} 0&0&1&0\\
0&0&0&1\\
{ - \displaystyle\frac{{{{k}_2} + {{k}_4}{{e}^{iq}}}}{{{{k}_1} + {{k}_3}{{e}^{iq}}}}}&0&0&{\displaystyle\frac{{\sum\nolimits_{j = 1}^4 {{{k}_j}} }}{{{{k}_1} + {{k}_3}{{e}^{iq}}}}}\\
0&{ - \displaystyle\frac{{{{k}_3} + {{k}_1}{{e}^{iq}}}}{{{{k}_4} + {{k}_2}{{e}^{iq}}}}}&{\displaystyle\frac{{{{e}^{iq}}\sum\nolimits_{j = 1}^4 {{{k}_j}} }}{{{{k}_4} + {{k}_2}{{e}^{iq}}}}}&0 \end{array}} \right]\left\{ \begin{array}{@{}l@{}} {\boldsymbol{p}}\\ \lambda {\boldsymbol{p}} \end{array} \right\} = \lambda \left( q \right)\left\{ \begin{array}{@{}l@{}} {\boldsymbol{p}}\\ \lambda {\boldsymbol{p}} \end{array} \right\}.
\end{eqnarray*}

The inner (outer) two bands with $| {{{\lambda }_{1,2}}} | < 1$ ($| {{{\lambda }_{3,4}}} | > 1$) correspond to the original (time-reversed) states, and we have ${{\lambda }_{3,4}}( q ) = \lambda _{1,2}^{ - 1}( { - q} )$ due to the PTS, with $P = I \otimes {{\sigma }_x}$ and $T = {{\sigma }_x} \otimes I$. The solid lines in Fig. 4b**–**d depict the inner bands of the periodic boundary condition (PBC) spectra, with their braid diagrams [51] shown in the insets. The PBC bands are symmetrically dispersed about the origin owing to the sublattice symmetry (SLS), $SH{{S}^{ - 1}} = - H$ with $S = {{\sigma }_z} \otimes {{\sigma }_z}$. Moreover, the decay spectrum forms a pair of exceptional points [52] (EPs) at $q = 0$ with ${{\lambda }_1} = {{\lambda }_3} = 1$ (red star) and ${{\lambda }_2} = {{\lambda }_4} = - 1$ (blue star). Specifically, $\lambda = 1$ corresponds to the uniform translation along the *n* direction (left panel in Fig. 4e), while $\lambda = - 1$ corresponds to the relative translation of two nested sublattices along the opposite directions and keeps the centroid unmoved (right panel in Fig. 4e). This is an interesting analog to the long acoustic and optical waves in diatomic crystals [24]. The open boundary condition (OBC) spectra are marked by the solid dots in Fig. 4b**–**d. All bulk modes (black dots) are localized at the boundaries due to the skin effect, as confirmed by the mean on-site amplitudes [53] shown in the top panel of Fig. 4f (green line). The decay length of the non-Bloch wave diverges at the EPs, $\lambda = \pm 1$, as characterized by the inverse participation ratio shown in Fig. 4g. These two points are denoted as the Bloch points [54], as the associated wavefunctions are delocalized in the bulk and correspond to the intersections of BZ and generalized BZ [55]. The Bloch points divide a pair of reciprocal skin modes [27,29,56] with the eigenvalues $\lambda $ and ${{\lambda }^{ - 1}}$ as well as oppositely biased spatial localizations, see the top panel of Fig. 4f. This is a nontrivial manifestation of the PTS. Moreover, the paired TZMs (red dots), where the applied load is completely blocked on the top boundary with negligible response inside the bulk, are pumped to the right edge (green line in the bottom panel of Fig. 4f), indicating a broken TRS (otherwise if the zero modes are equally localized at the two boundaries as in Hermitian systems [26], the IS and thereby TRS must be preserved owing to the PTS). Accordingly, another set of TZMs with $\lambda = \infty $ and a left localization is ensured by the PTS (blue line), where the load applied on the bottom boundary is blocked. We also note that when ${{k}_1}{{k}_4} = {{k}_2}{{k}_3}$, the rhombus lattice is ‘net-reciprocal’ for the entirely real spectrum. In this case, the incident wave packet forms a standing wave even if the lattice has no symmetries, as detailed in Supplementary Section VIII.

**Figure 5.**
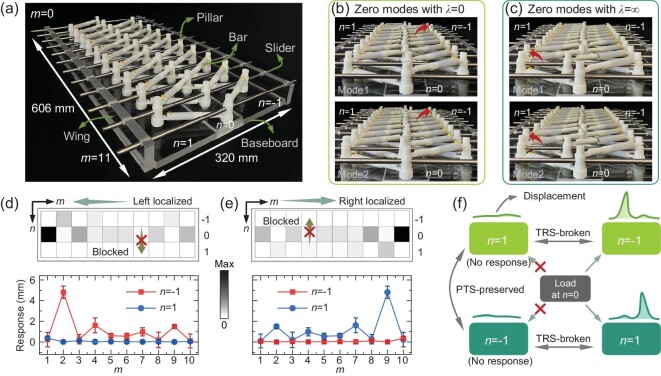
Observation of the broken TRS and PTS-protected reciprocal TZMs. (a) Side view of the assembled truss-like lattice with a single DoF and the size $M \times N = 10 \times 3$. The stiffnesses are $( {{{k}_1},{{k}_2},{{k}_3},{{k}_4}} ) = ( {2,2,3,6} )$. (b and c) Deformation plots under the paired TZMs (Mode 1 and Mode 2) with $\lambda = 0$ (b) and $\lambda = \infty $ (c), respectively. Sites having the largest displacements are marked by the red arrows. (d and e) The top panels show the simulated deformation fields under the applied TZMs with $\lambda = 0$ (d) and $\lambda = \infty $ (e). The experimental measurements are shown in the bottom panels as error bars. (f) Illustration of the symmetry identification in terms of the TZM. The original (time-reversed) states with $\lambda = 0$ ($\lambda = \infty $) and a left (right) boundary localization induce a strong response at $n = - 1$ ($n = 1$) and neglected response at $n = 1$ ($n = - 1$), implying that the TRS is broken and the PTS is preserved.

The broken TRS is experimentally demonstrated in an assembled truss-like lattice shown in Fig. 5a (Supplementary Section XIII). The load that corresponds to the eigenstate of the TZM ($\lambda = 0$ or $\lambda = \infty $) is applied on the middle row ($n = 0$). The responses at $n = 1$ and $n = - 1$ are, respectively, the original and time-reversed states. Because TRS refers to the *n*-reversal symmetry, it means that the deformation must be blocked at both $n = \pm 1$ under the zero modes. However, the measured results shown in Fig. 5b**–**e indicate that only $n = 1$ ($n = - 1$) enjoys deformation shielding under the applied zero modes $\lambda = 0$ ($\lambda = \infty $) with spatial localization at $m = 1$ ($m = 10$), while a strong response is observed for another row, $n = - 1$ ($n = 1$). These results demonstrate that the TRS is broken and the PTS is preserved, as pictorially illustrated in Fig. 5f, see also Supplementary Section XIII. It should be noted that in this experiment, the diagonal bar stiffness is assumed to be constant and any possible nonlinear effects have been neglected. This assumption is reasonable, as can be seen in Supplementary Section XIII. In this sense, the synergy of zero modes, skin effect, and band topology facilitates selective deformation shielding that is space-time correlated, i.e. loads exerted on opposite spatial biases induce deformation blockages along opposite temporal biases, which might be utilized for eccentric load shielding.

## DISCUSSION

In summary, we have unraveled the space-time duality between wave dynamic systems and higher-dimensional static systems and proposed a general framework for characterizing localized static deformation responses in mechanical metamaterials via non-Hermitian band theory. The TRS is broken in asymmetric lattices, which induces unidirectional stress guiding on a designated path. Steering the symmetry of these building blocks enables the construction of stress guides in assembled heterostructures, where the stress fields are directionally accumulated and the localized static deformation mode can be tailored. A reciprocal TZM supported by the intrinsic PTS is verified, where loads localized at different boundaries induce deformation blockages along the opposite transverse directions. Because our theory is based on band structures, it is accessible to mechanical metamaterials with periodic microstructures. Such broader ranges of stress guides without further restrictions of material microstructures and external driving forces can facilitate their wide applications in stress cloaking [12], energy harvesting [13], and information processing devices [14,15].

## Supplementary Material

nwae110_Supplemental_File
