# Supplementary material for: Stress guides in generic static mechanical metamaterials
Source: Natl Sci Rev. 2024 Mar 22;11(9):nwae110. doi: 10.1093/nsr/nwae110 (PMC11321258; doi:10.1093/nsr/nwae110)
Supplement: nwae110_Supplemental_File [file nwae110_supplemental_file.pdf]

Supplementary data for

Stress guides in generic static mechanical metamaterials

Aoxi Wang and Chang Qing Chen *

Department of Engineering Mechanics, CNMM and AML, Tsinghua University

Beijing 100084, PR China

*To whom correspondence should be addressed;

E-mail: chencq@tsinghua.edu.cn

I. Mechanical analogue of the Hatano-Nelson model

II. Underlying symmetries in the mechanical HN model and SR model

III. Correspondence between 1D D'Alembert equation and Lame's equation

IV. Discretization of Lame's equation

V. Group velocity of the wave packet-formed deformation mode

VI. Static lattices with released degrees of freedom and random couplings

VII. Generality of the transmission theory for static deformation fields

VIII. Mechanical analogue of the non-Hermitian SSH model

IX. 3D static Rayleigh model

X. Comparison of topological matters in synthetic dimensions

XI. Hunting the low-energy physics in static Rayleigh model

XII. Numerical simulations

XIII. Experimental implementations

References

* Corresponding author, chencq@tsinghua.edu.cn.

I. Mechanical analogue of the Hatano-Nelson model

1. Bloch Hamiltonian

Originally proposed for studying the localization transition in non-Hermitian quantum mechanics [1,2], the Hatano-Nelson (HN) model represents a 1D monoatomic chain with non-reciprocal hoppings and random on-site potentials (defects). A mechanical realization of the clean HN model without defects is a monoatomic mass-spring chain with asymmetric coupling stiffnesses, $k_{\mathrm{L}\to\mathrm{R}} = k_1$ and $k_{\mathrm{R}\to\mathrm{L}} = k_2$, see Fig. S1a. In real space, the dynamic equation of the mechanical HN chain with homogeneous mass density μ is $\ddot{\boldsymbol{u}}(t) + \boldsymbol{D}\boldsymbol{u}(t) = 0$, where $\boldsymbol{u}$ is the displacement vector and $\boldsymbol{D} = \boldsymbol{K}/\mu$ is the asymmetric dynamic matrix (assume $\mu = 1$ in the following). Applying the time-harmonic solution $\boldsymbol{u}(t) = e^{-i\omega t}\boldsymbol{\psi}$ with ω the angular frequency and $\boldsymbol{\psi}$ the eigen-modal, the dynamic equation can be reformulated as the following eigen-equation

$$\omega \begin{Bmatrix} \boldsymbol{u}(0) \\ \dot{\boldsymbol{u}}(0) \end{Bmatrix} = i \begin{bmatrix} 0 & 1 \\ -\boldsymbol{D} & 0 \end{bmatrix} \begin{Bmatrix} \boldsymbol{u}(0) \\ \dot{\boldsymbol{u}}(0) \end{Bmatrix} \tag{S1}$$

where $\boldsymbol{u}(0) = \boldsymbol{\psi}$ and $\dot{\boldsymbol{u}}(0)$ is the initial velocity. Under PBC, the displacement and velocity vectors can be Fourier transformed, i.e., $\boldsymbol{u}(0) = \frac{1}{\sqrt{M}} \sum_{q'} u(q')\boldsymbol{W}(q')$, $\dot{\boldsymbol{u}}(0) = \frac{1}{\sqrt{M}} \sum_{q'} v(q')\boldsymbol{W}(q')$, with *M* being the number of lattice sites and $W_m(q) = e^{iqm}$ the components of Fourier base vectors, respectively, and $u(q)$ and $v(q)$ are the corresponding Fourier coefficients. Then, Eq. (S1) can be expressed as

$$\omega \sum_{q'} \begin{Bmatrix} u(q') \\ v(q') \end{Bmatrix} \otimes \boldsymbol{W}(q') = i \begin{bmatrix} 0 & 1 \\ -\boldsymbol{D} & 0 \end{bmatrix} \sum_{q'} \begin{Bmatrix} u(q') \\ v(q') \end{Bmatrix} \otimes \boldsymbol{W}(q') \tag{S2}$$

Using the orthonormality of the base vectors, $\langle \boldsymbol{W}(q') | \boldsymbol{W}(q) \rangle = M\delta_{qq'}$, and noting that $v(q) = -i\omega u(q)$, we attain the following eigen-equation in the momentum space by left-dotting $(1,1) \otimes \langle \boldsymbol{W}(q)|$ to both sides of Eq. (S2)

$$\omega\begin{Bmatrix} u(q) \\ v(q) \end{Bmatrix} = i\begin{bmatrix} 0 & 1 \\ k_1 e^{-iq} + k_2 e^{iq} - k_1 - k_2 & 0 \end{bmatrix}\begin{Bmatrix} u(q) \\ v(q) \end{Bmatrix} \tag{S3}$$

where we have used the relation, $\left\langle \boldsymbol{W}\left(q^{'}\right) \middle| \boldsymbol{DW}(q) \right\rangle = M\left(k_1 + k_2 - k_1 e^{-iq} - k_2 e^{iq}\right)\delta_{qq^{'}}$. The above equation yields a factorized Bloch Hamiltonian of the HN model, $H_{\mathrm{HN}}(q) = a\sigma_x - ib\sigma_y$ with $a(b) = i\left(k_1 e^{-iq} + k_2 e^{iq} \pm 1 - k_1 - k_2\right)/2$. When $k_1 = k_2 = k$, we can also make a similarity transformation [3] $TH_{\mathrm{HN}}T^{-1} = h$ with $T = \mathrm{diag}\{\sqrt{2k\left(1-\cos(q)\right)},\ i\}$ to restore the Hermiticity of the Hamiltonian, $h = h^{\dagger}$.

2. Correspondence to quantum mechanics

As mentioned in the main text, the continuous wave equation of the HN chain is $\partial_t^2 u_m - c^2\partial_m^2 u_m + 2\varepsilon c^2 \partial_m u_m / h = 0$, with $c = h\sqrt{k/\mu}$, $\varepsilon = \left(k_2 - k_1\right)/\left(k_2 + k_1\right)$ and *h* being respectively the sound velocity, the degree of non-reciprocity, and the lattice constant. Although it is possible to map this wave equation to the Schrödinger form, which is first-order in time, the time-harmonic assumption makes this technique somewhat a priori [4]. Here we consider the Klein-Gordon equation in relativistic quantum mechanics which is intrinsically second-order in time, $-\psi_{,tt} = \left(-\nabla^2 + 1\right)\psi$ for a free particle in the scaled form ($\hbar = m = c = e = 1$). For a 1D system under a uniform vector potential, we have $-\psi_{,tt} = \left[\left(-i\partial_m + A\right)^2 + 1\right]\psi$. After eliminating the constant terms that shift the whole energy band and are irrelevant to the state topology, we arrive at $\partial_t^2\psi - \partial_m^2\psi - 2iA\partial_m\psi = 0$, which can be mapped to the continuous HN model under an imaginary gauge potential, $iA \in \mathbb{R}$. It is known that the Klein-Gordon equation can be degraded to the Schrodinger equation in the nonrelativistic limit, and the latter, under the imaginary gauge field, captures the non-Bloch characters of the wavefunctions (i.e., the skin effect) [5]. Also, taking a square root of the former Klein-Gordon equation, one obtains a first-order non-Hermitian Dirac equation resulted from the violation of Lorentz symmetry, and the skin effect emerges in such a system [6].

II. Underlying symmetries in the mechanical HN model and SR model

For the mechanical HN model, the time-reversed state under the flip $t \to -t$ is $u_m^T(t) = u_m(-t) = e^{i\omega t}e^{iqm}$, for which the time-reverse symmetry (TRS) is preserved only if $q(\omega) = q(-\omega)$. Note that in classical mechanical systems all physical quantities (e.g., displacement, momentum, and angular momentum) are real-valued, so the complex conjugation in the time-reversal process does not affect any physical observables compared to the quantum mechanical counterpart. Indeed, the time-reversed state at time $-t$ (i.e., $u_m^T(-t)$) has the same real-space coordinate and an opposite momentum compared with the original state at time t (i.e., $u_m(t)$), which is a basic requirement for time-reversal. The TRS is preserved in the HN model, $TH_{\mathrm{HN}}(q)T^{-1} = -H_{\mathrm{HN}}(q)$ with $T = \sigma_z$, as it only possesses non-reciprocal couplings acting on the space dimension while no TRS broken terms (such as the Coriolis force and gyroscopic force) are invoked. This unitary symmetry is also referred to as the sublattice symmetry (SLS) for bipartite lattices [7]. The non-reciprocal couplings inject additional energy to a preferred direction toward which the wave packet is amplified, while it is attenuated along the opposite direction due to the extraction of vibration energy from the environment. This directional amplification emulates a chiral wave transport in TRS broken systems [8].

Furthermore, both the HN model and the static Rayleigh (SR) model sustain an antiunitary τ -symmetry, $\tau H_{\mathrm{HN/SR}}(q)\tau^{-1} = H_{\mathrm{HN/SR}}(-q)$ with $\tau = \sigma_z K / K$ and K the complex conjugation. The τ -symmetry actually corresponds to the conventional TRS in quantum mechanics [9]. It should be noted that in the HN chain, the τ -symmetry is exactly the same as the unitary TRS defined above, since they are correlated with each other due to the intrinsic particle-hole symmetry (PHS) protection [3,4], $CH_{\mathrm{HN}}(q)C^{-1} = -H_{\mathrm{HN}}(-q)$ with $C = K$ a combination of T and τ. In addition to this bosonic TRS, there are also attempts to artificially construct a fermionic TRS for emulating the Kramers degeneracy in mechanical systems [10]. In our SR model, on the other hand, the combination of intrinsic parity-time symmetry (PTS) and τ -symmetry yields another antiunitary symmetry, $\Gamma H_{\mathrm{SR}}(q)\Gamma^{-1} = H_{\mathrm{SR}}^{-1}(q)$ with

$\Gamma = \sigma_x K$, giving rise to $q(\eta) = q(-\eta^*)$. Interestingly, the PTS and Γ symmetry have the same effect on the eigen-spectrum compared to the transposition version of the non-Hermitian PHS and chiral symmetry (CS) [7], respectively. By contrast, a conjugation version of the PHS in the HN model is denoted as $\text{PHS}^\dagger$. Consequently, the combination of $\text{PHS}^\dagger$ and τ -symmetry leads to the SLS in the HN model, giving rise to preserved TRS. While the joint PHS and τ -symmetry in our static system leads to the CS, which does not coincide with the physical TRS as long as η is complex, as illustrated in Fig. S1.

III. Correspondence between 1D D'Alembert equation and Lame's equation

The equilibrium conditions of a 2D isotropic elastic continuum under the state of plane-strain are governed by the following Lame's equation [11]

$$\begin{cases} 2(1-\nu)\dfrac{\partial^2 u_m(m,n)}{\partial m^2} + \dfrac{\partial^2 u_n(m,n)}{\partial m \partial n} + (1-2\nu)\dfrac{\partial^2 u_m(m,n)}{\partial n^2} = 0 \\ (1-2\nu)\dfrac{\partial^2 u_n(m,n)}{\partial m^2} + \dfrac{\partial^2 u_m(m,n)}{\partial m \partial n} + 2(1-\nu)\dfrac{\partial^2 u_n(m,n)}{\partial n^2} = 0 \end{cases} \tag{S4}$$

Where ν is Poisson's ratio. The in-plane (i.e., $m-n$ plane) displacement fields along the horizontal and vertical directions are $u_m(m,n)$ and $u_n(m,n)$, respectively, while the out-of-plane deformation is zero. Because the 1D D'Alembert equation is acted on a scalar field, we take the hypothesis $u_m(m,n) = Cu_n(m,n)$ with a (constant) complex coefficient C to eliminate the redundant component. Note that the hypothesis only accounts for a special solution of Lame's equation and can be extracted from the other deformation modes by applying a delicate boundary condition. Applying the hypothesis to Eq. (S4) and linearly combining the two equations to eliminate the mixed partial derivative (i.e., $\partial^2 u_{m/n} / \partial m \partial n$), we thereafter obtain Eq. (1) in the main text, which is in a form similar to the 1D D'Alembert equation under an imaginary transformation, $n = it$. By employing the SR solution ($u_n(m,n) = e^{-\eta n} e^{iqm}$) and plugging it into Eq. (S4), we obtain a linear dispersion $\eta(q) = \pm q$ along with the coefficient $C = \mp i$. These results are irrelevant to the material property (i.e., Poisson's ratio), since the displacement field of the sinusoidal SR mode is incompressible.

IV. Discretization of Lame's equation

By dividing the $m-n$ plane into a series of equally spaced square meshes with the step length *h*, the 2D continuum is discretized as a planar square lattice. The nodal coordinates are mh and nh with *m* and *n* being the node labels along the horizontal and vertical directions, respectively. As a result, the second order partial differentials in Eq. (1) can be recast as the linear combination of discrete nodal displacements via the finite difference method [12], e.g., $\partial^2 u_n / \partial m^2 = \left(u_{m+1,n} + u_{m-1,n} - 2u_{m,n}\right) / h^2$. Substituting it into Eq. (1), the following algebraic equation can be obtained

$$u_{m,n-1} + u_{m,n+1} + \frac{(1-2\upsilon) - 2C^2(1-\upsilon)}{2(1-\nu) - C^2(1-2\nu)}\left(u_{m-1,n} + u_{m+1,n}\right) = 2\left(1 + \frac{(1-2\upsilon) - 2C^2(1-\upsilon)}{2(1-\nu) - C^2(1-2\nu)}\right)u_{m,n} \tag{S5}$$

By contrast, the quasi-static equilibrium equation for the interior ($n > 0$) of the symmetric X-braced lattice with equal diagonal stiffnesses $k_1 = k_2$ and a single degree-of-freedom (DoF) is given by

$$u_{m,n-1} + u_{m,n+1} + u_{m-1,n} + u_{m+1,n} + k_1 \sum u_{m\pm1,n\pm1} = k_\mathrm{t} u_{m,n} \tag{S6}$$

with the summation running over all superscripts and $k_\mathrm{t} = 4 + 4k_1$ denoting the total stiffness of a lattice node. Evidently, Eq. (S6) asymptotically approaches Eq. (S5) when $k_1 \to 0$, since we have $\frac{(1-2\upsilon) - 2C^2(1-\upsilon)}{2(1-\nu) - C^2(1-2\nu)} = 1$ ($C = \mp i$) for an isotropic continuum. The correspondence between the lattice and the continuum implies that the bulk displacement field of X-braced lattices can be selected as the SR form, which provide a practically feasible approach for implementing the delicate boundary condition, as corroborated in Fig. 5.

V. Group velocity of the wave packet-formed deformation mode

The mathematical equivalence between the 1D D'Alembert equation and 2D Lame's equation, as well as their analogous displacement solutions, render it natural to explore the "dynamic" transport property in our static model, with the state evolution of an input signal taken along the spatial *n* direction. Consider a quasi-static

displacement load applied on the top boundary $n=0$ with a random form, $u_{m,0}$. It can be expanded as a series of harmonic components by Fourier transform, $u_{m,0}=\sum_q f(q)e^{iqm}/M$, where the wavenumber is $q=2\pi l/M$ with M the site number along the m axis and integer l. The Fourier coefficient is $f(q)=\sum_m u_{m,0}e^{-iqm}$. Then, the excited bulk response ($n>0$) is a linear combination of the SR modes associated with those sinusoidal boundary loads

$$u_{m,n}=\frac{1}{M}\sum_q f(q)e^{-\eta(q)n}e^{iqm} \tag{S7}$$

Suppose $u_{m,0}$ is centered at a wavenumber q_0 and the width of the wavenumber window is narrow, $\delta q \ll 1$, emulating a wave packet [13] prepared at its initial state ($t=0$). Then, the summation in Eq. (S7) can be reduced as

$$u_{m,n}=\frac{1}{M}\sum_{q=q_0-\delta q}^{q_0+\delta q} f(q_0)e^{-\eta(q)n}e^{iqm} \tag{S8}$$

where we have neglected the slight variation of $f(q)$ in the summation interval. Let $\xi=q-q_0$ being the deviation of the wavenumber away from the center, we have

$$u_{m,n}=\frac{1}{M}\sum_{\zeta=-\delta q}^{\delta q} f(q_0)e^{-\eta(\zeta+q_0)n}e^{i(\zeta+q_0)m} \tag{S9}$$

When both the real and imaginary parts of the decay spectrum at q_0 is smooth, e.g., in the long wavelength limit, we can expand the decay factor to the linear order, $\eta(\zeta+q_0)=\eta(q_0)+\eta^{'}(q_0)\zeta$, yielding

$$u_{m,n}=\frac{f(q_0)e^{iq_0m}e^{-\eta(q_0)n}}{2\pi}\sum_{\zeta=-\delta q}^{\delta q} e^{-\eta^{'}(q_0)\zeta n}e^{i\zeta m}\Delta\zeta \tag{S10}$$

with $\Delta\zeta=2\pi/M$. For a sufficiently large system with $M\gg 1$, the summation in Eq. (S10) can be approximated as a continuous integration of ξ, that is

$$u_{m,n}=\frac{f(q_0)e^{iq_0m}e^{-\eta(q_0)n}}{2\pi}\int_{-\delta q}^{\delta q} e^{-\eta^{'}(q_0)\zeta n}e^{i\zeta m}d\zeta \tag{S11}$$

and its solution is

$$u_{m,n}=\frac{f(q_0)\delta q}{\pi}\frac{\sin(\gamma)}{\gamma}e^{-a(q_0)n}e^{i(q_0m-b(q_0)n)} \tag{S12}$$

with $\gamma(n,m)=(m-b^{'}(q_0)n)\delta q+a^{'}(q_0)n\delta qi$ and the complex expanded decay factor, $\eta(q)=a(q)+b(q)i$. The term $e^{i(q_0m-b(q_0)n)}$ in $u_{m,n}$ is the phase factor that controls the fine structure of the wave packet, while $e^{-a(q_0)n}$ is a global decay factor originated from the attenuation nature of the boundary load (i.e., the Saint-Venant's edge effect) and has no effect on the local property of the deformation mode during its penetration in the bulk. As a result, the local amplitude of the wave packet at a given coordinate n is determined by $\sin(\gamma)/\gamma$, and its square modulus is given by

$$\left|\frac{\sin(\gamma)}{\gamma}\right|^2=\frac{e^{2\mathrm{Im}(\gamma)}+e^{-2\mathrm{Im}(\gamma)}+2\sin^2(\mathrm{Re}(\gamma))-2\cos^2(\mathrm{Re}(\gamma))}{4\mathrm{Re}^2(\gamma)+4\mathrm{Im}^2(\gamma)} \tag{S13}$$

We first neglect the term $\mathrm{Im}(\gamma)$, for which the above equation can be simplified as

$$\left|\frac{\sin(\gamma)}{\gamma}\right|^2\sim\frac{2(1-\cos(2\mathrm{Re}(\gamma)))}{4\mathrm{Re}^2(\gamma)}=\left(\frac{\sin(\mathrm{Re}(\gamma))}{\mathrm{Re}(\gamma)}\right)^2 \tag{S14}$$

This function takes its maximum at $\mathrm{Re}(\gamma)=0$. As a result, the *m*-directional location of the wave packet peak is determined by $m_{\max}=b^{'}(q_0)n$. Furthermore, the changing rate of the peak with respect to the *n* axis characterizes the skewing velocity of the wave packet during its spatial evolution and is defined as the group velocity, i.e., $v_g(q)=dm_{\max}/dn=d\,\mathrm{Im}(\eta)/dq$, as detailed in the main text. This is a natural extension from wave dynamics since the imaginary gauge transform ($n=it$) switches the roles of real and imaginary decay spectra compared with the vibration spectrum.

More rigorously, if we retain the term $\mathrm{Im}(\gamma)$ in Eq. (S13) and expand the exponents in the numerator to the second order, we have

$$\left|\frac{\sin(\gamma)}{\gamma}\right|^2\sim\frac{\sin^2(\mathrm{Re}(\gamma))+\mathrm{Im}^2(\gamma)}{\mathrm{Re}^2(\gamma)+\mathrm{Im}^2(\gamma)} \tag{S15}$$

Because $\sin^2\left(\mathrm{Re}(\gamma)\right) \le \mathrm{Re}^2(\gamma)$, the maximum of Eq. (S15) is still at $\mathrm{Re}(\gamma)=0$, yielding the group velocity definition. Besides, the curve in Eq. (S15) as a function of *m* (i.e., the wave packet envelope) is flattened when $\mathrm{Im}(\gamma)$ increases, as illustrated in Fig. S2a for a symmetric X-braced lattice with $b'(q_0)=0$. This implies that the incident wave packet is inevitably broadened when penetrating the bulk, and the larger the gradient of the real decay spectrum (i.e., $a'(q_0)$) is, the more the wave packet is flattened. This, accompanied by the global decay factor $e^{-a(q_0)n}$, is the major reason why the incident wave-packet-formed static deformation mode decays and collapses during its spatial evolution.

One thing that should be concerned about is that when $q_0=0$, the coefficients of the linear order in the expanded decay factor are $\eta'(0^-)$ and $\eta'(0^+)$ for $\zeta<0$ and $\zeta>0$, respectively, to ensure the positivity of $\mathrm{Re}(\eta)$. In this sense, Eq. (S11) should be amended as (discarding the constant coefficient)

$$u_{m,n} \sim \int_{-\delta q}^{0^-} e^{-\eta'(0^-)\zeta n} e^{i\zeta m} d\zeta + \int_{0^+}^{\delta q} e^{-\eta'(0^+)\zeta n} e^{i\zeta m} d\zeta \tag{S16}$$

which can be solved as

$$u_{m,n} \sim \frac{1}{s} + \frac{1}{s^*} - e^{-a'(q_0)\delta q n}\left(\frac{e^{i\delta q\left(m-b'(q_0)n\right)}}{s^*} + \frac{e^{-i\delta q\left(m-b'(q_0)n\right)}}{s}\right) \tag{S17}$$

where $s(m,n)=a'(q_0)n+\left(m-b'(q_0)n\right)i$. Eq. (S17) can be further simplified as

$$u_{m,n} \sim 2\,\mathrm{Re}\left(\frac{1}{s}\right) - 2e^{-a'(q_0)\delta q n}\,\mathrm{Re}\left(\frac{e^{-i\delta q\left(m-b'(q_0)n\right)}}{s}\right) \tag{S18}$$

It can be seen that the second term in Eq. (S18) decreases exponentially faster compared with the first term, so it can be neglected in the deep bulk. Accordingly, the local maximum of the first term in Eq. (S18) at a given coordinate *n* is arrived only when $m-b'(q_0)n=0$ is satisfied. This suggests that the group velocity at $q_0=0$ can still be formulated as $v_g(q)=d\,\mathrm{Im}(\eta)/dq$, and the spatial evolution of the wave

packet peak is scaled with $\left(a'(q_0)n\right)^{-1}$, which decays slower compared with the exponential attenuation at $q_0 \neq 0$ (c.f., Eq. (S12)). Furthermore, at the neighbor of $m_{\max}$, e.g., at $m = m_{\max} \pm \Delta m$, we have $s(m,n) = a'(q_0)n \pm \Delta m i$. Then, the ratio of $u_{m,n}$ and $u_{m_{\max},n}$ is given by

$$\frac{u_{m,n}}{u_{m_{\max},n}} = \mathrm{Re}\left(\frac{1}{s(m,n)}\right) \Big/ \mathrm{Re}\left(\frac{1}{s(m_{\max},n)}\right) = 1 - \frac{\Delta m^2}{a'(q_0)^2 n^2 + \Delta m^2} \qquad \text{(S19)}$$

Evidently, for given n and Δm, the envelope of the wave packet becomes flatter (i.e., $u_{m,n}/u_{m_{\max},n} \to 1$) when $a'(q_0)$ is enlarged, which conforms with the above analysis at $q_0 \neq 0$.

On this basis, we can alleviate wave packet distortion and construct a high-quality stress guide by reducing the gradient of the real decay spectrum of a metamaterial. As an illustration, Fig. S2b depicts the gradients of the real decay spectrum at the center wavenumber $q_0 = 0$ in symmetric X-braced lattices (i.e., $k_1 = k_2 = k$). It can be seen that the gradient is suppressed when *k* decreases, for which we expect a higher concentration and lower attenuation of the static deformation field in the middle bulk region since $v_g = 0$. This is visualized in Fig. S2c, where a high fidelity of stress localization is observed when *k* is small, while the deformation fields are distorted for large *k*, consistent with our analysis.

The group velocity cannot only help us predict the static deformation pattern within the lattice material under an applied boundary load, but also facilitate the exploration of (time-dependent) dynamic evolution of a non-reciprocal wave pulse in our static system based upon purely passive modulation. In addition, the reason we choose a concentrated point load to excite the wave packet with a center wavenumber at $q_0 = 0$ is not only due to its simplified implementation, but also for the sake of better visualization of the deformation response in the deep bulk of the lattice, benefited from the small wave packet attenuation rate at $q_0 = 0$.

VI. Static lattices with released degrees-of-freedom and random couplings

1. Two degrees-of-freedom per node

The Bloch Hamiltonian of the X-braced lattice with two DoFs per node can be expressed as (the derivation can be found in refs. [11,14,15], see also Section VIII for a similar four band model)

$$H(q)=\begin{bmatrix} \mathbf{0} & I \\ -\boldsymbol{K}_{-1}^{-1}(q)\boldsymbol{K}_{1}(q) & \boldsymbol{K}_{-1}^{-1}(q)\boldsymbol{K}_{0}(q) \end{bmatrix} \quad \text{(S20)}$$

where $\mathbf{0}$ and I respectively denote the 2×2 zero matrix and identity matrix, and $\boldsymbol{K}_i(q)=\sum_{j=-1}^{1}\boldsymbol{k}_{ji}e^{-iqj}$ with $i=-1,\ 0,\ 1$. The stiffness matrices are defined as

$$\boldsymbol{k}_{01}=\boldsymbol{k}_{0-1}=\begin{bmatrix} 1 & 0 \\ 0 & 0 \end{bmatrix} \quad \boldsymbol{k}_{00}=\begin{bmatrix} 2(k_1+k_2)\cos^3(\theta)+2 & 2(k_1-k_2)\cos^2(\theta)\sin(\theta) \\ 2(k_1-k_2)\cos^2(\theta)\sin(\theta) & 2(k_1+k_2)\cos(\theta)\sin^2(\theta) \end{bmatrix}$$

$$\boldsymbol{k}_{1-1}=\boldsymbol{k}_{-11}=\begin{bmatrix} k_2\cos^3(\theta) & -k_2\cos^2(\theta)\sin(\theta) \\ -k_2\cos^2(\theta)\sin(\theta) & k_2\cos(\theta)\sin^2(\theta) \end{bmatrix}$$

$$\boldsymbol{k}_{11}=\boldsymbol{k}_{-1-1}=\begin{bmatrix} k_1\cos^3(\theta) & k_1\cos^2(\theta)\sin(\theta) \\ k_1\cos^2(\theta)\sin(\theta) & k_1\cos(\theta)\sin^2(\theta) \end{bmatrix} \quad \text{(S21)}$$

Here, $k_{1/2}=(EA)_{1/2}/(EA)_0$ refers to the relative tensile modulus between the diagonal and vertical bars, while for single DoF cases, $k_{1/2}$ is defined as the *n*-directional reaction force of the linking bars induced by the unit *n*-directional translation of two connected nodes. θ denotes the inclination angle of the diagonal bars with respect to the *n* axis (we set $\theta=\pi/4$ in this study). The element in the stiffness matrix relates the displacement component of a driving node and the resulting central force exerted on a receiving node. For example, the element $[\boldsymbol{k}_{ij}]_{21}$ denotes the vertical component of the exerted force on node (m,n) induced by a unit horizontal displacement of node $(m-i,n-j)$. We have discarded the horizontal internodal interactions (so that $\boldsymbol{k}_{10}=\boldsymbol{k}_{-10}=0$), which is also the case for numerical simulations of the single DoF X-braced lattices, since the horizontal bars cannot generate any coupling force when the

node can only translate vertically with infinitesimal magnitudes. The Hamiltonian in Eq. (S20) is also referred to as the displacement transfer matrix [16], with its eigenvalue being the decay factor $\lambda = e^{-\eta}$ and $\boldsymbol{\psi} = \{\boldsymbol{p}, \lambda\boldsymbol{p}\}^T$ the eigenstate, while $\boldsymbol{p} = \{p_m, p_n\}^T$ is the polarization vector of the lattice node.

We focus on the lattice symmetry inherited in the Hamiltonian. For the single DoF case (i.e., Eq. (2)), the space inversion requires that $q \to -q$ and $\lambda \to \lambda$, for which the inversion operator converts the momentum but has no effect on the eigenstate (i.e., $\boldsymbol{\psi} = \{1,\ \lambda\}^T$), and is therefore selected as the identity matrix. In this sense, we have $H(-q)I\boldsymbol{\psi}(q) = \lambda(q)I\boldsymbol{\psi}(q)$ if the inversion symmetry (IS) is preserved, yielding the symmetry definition in the main text. The time-reverse, on the other hand, requires that $q \to q$ and $\lambda \to -\lambda$, and its action on the eigenstate gives that $T\boldsymbol{\psi} = \{1,\ \lambda^{-1}\}^T \sim \{\lambda,\ 1\}^T$ up to a coefficient. An appropriate choice of this TRS operator is the Pauli matrix, σ_x, which yields that $H(q)T\boldsymbol{\psi}(q) = \lambda^{-1}(q)T\boldsymbol{\psi}(q)$ and thereby the symmetry definition. By contrast, the space inversion for the two DoFs lattices, which is taken as the mirror-reflection along the *m* axis, flips p_m in the polarization vector while keeps p_n unchanged. This suggests us to select $P = I \otimes \sigma_z$. The time-reverse is taken as the mirror-reflection along the *n* axis and only flips p_n (as well as $\lambda \to \lambda^{-1}$), and a proper choice is $T = \sigma_x \otimes \sigma_z$. All these symmetries are preserved in the reciprocal lattices with $k_1 = k_2$, while broken for $k_1 \neq k_2$.

The decay spectra with two sets of stiffness parameters are shown in the bottom panels of Fig. 1f and g. The four bands degenerate at the origin when $q = 0$ and form a pair of (second-order) exceptional points with only two independent eigenvectors, namely, $\boldsymbol{p} = \{0,\ 1\}^T$ and $\{1,\ 0\}^T$, corresponding to the trivial translations along the vertical and horizontal directions, respectively. On the other hand, the middle two bands degenerate at $q = \pm\pi$ with $\eta = \pm\pi i$ (i.e., $\lambda = -1$) and form a new exceptional point, which is responsible for the floppy deformation mode of the lattice (i.e., a set of nodal displacements that leads to no energy storage in the bar elements [17]) under the infinitesimal strain field. This floppy mode is absent in the single DoF case, but

the latter instead provides a venue for pursuing SR model-based low-energy physics, as detailed in Section XI.

Furthermore, $\mathrm{Im}(\eta)$ is symmetrically dispersed with respect to the center of the Brillouin zone (BZ) when $k_1 = k_2$, see the lower panel of Fig. 1f, implying the preservation of TRS along with a bidirectional transport of the wave packet, as shown in the top and middle panels of Fig. 1f. By contrast, $\mathrm{Im}(\eta)$ disperses asymmetrically in the non-reciprocal lattice ($k_1 \neq k_2$) owing to the broken TRS (lower panel, Fig. 1g). In particular, the slopes of the two branches have opposite signs and unequal magnitudes nearing the BZ center, indicating that the wave packet induced by a point load possesses different group velocities when traveling along the opposite directions, thereby forming an asymmetric bidirectional stress guide. The upper halves of the top and middle panels of Fig. 1g shows the simulated and measured displacement fields for the original state with $\mathrm{Re}(\eta) > 0$, and we indeed observe two wave packets propagating along opposite directions with different group velocities. For the time-reversed state where a point load is applied on the bottom boundary with $\mathrm{Re}(\eta) < 0$, the asymmetric bifurcations are still observed with an identical deformation distribution with respect to the original state (lower halves) due to the PTS protection. Specifically, this arises from the fact that the original state and the time-reversed state share the same group velocity (the imaginary decay spectrum is doubly degenerate for $\pm\mathrm{Re}(\eta)$), with one branch propagating (either amplified or attenuated) from the top-right corner to the bottom-left corner having a larger group velocity, and another branch propagating from the top-left corner to the bottom-right corner having a smaller group velocity (recall that the positive evolution direction is taken along the $+n$ axis). This bidirectional stress localization is in sharp contrast with single DoF cases, where $\mathrm{Im}(\eta)$ disperses monotonously and only a unidirectionally propagated mode can be excited, as identified in the main text.

2. *Random coupling effects*

The X-braced lattice considered above is the simplest model where (passive) non-reciprocity can be invoked. For a general planar lattice with a square grid and a

random set of internodal couplings (Fig. S3a, consider single DoF), the eigen-equation has the same form as Eq. (2) and the matrix elements of the Hamiltonian, based on Eq. (S20), can be expressed as

$$\begin{aligned} K_0(q) &= 2 + 2\sum_{s=1}^{\beta}\left(k_{s,1} + k_{s,2}\right) \\ K_1(q) &= K_{-1}(-q) = 1 + \sum_{s=1}^{\beta}\left(k_{s,1}e^{iqs} + k_{s,2}e^{-iqs}\right) \end{aligned} \tag{S22}$$

where $k_{s,1}$ and $k_{s,2}$ respectively denote the top-left to bottom-right (blue bars) and top-right to bottom-left (green bars) sectors of the diagonal bar stiffness for the *s*th-order nearest-neighbor couplings, with β being the degree of non-locality, see Fig. S3a for a schematic of the general configuration of the associate cell, i.e., the smallest unit containing all interactions of a node [14]. For example, $k_{1,1} = k_1$ and $k_{1,2} = k_2$ for X-braced lattices with $\beta = 1$ (Fig. 1a), and $k_{2,1} = k_3$ and $k_{2,2} = k_4$ for modified X-braced lattices with $\beta = 2$ (Fig. 3a). The vertical stiffness is unitized by convention (solid gray bars) and the horizontal stiffness has been neglected (dashed gray lines). Different from the two DoFs case (i.e., Eq. (S20)), the Fourier transformed stiffness matrix is now in scalar form, i.e., Eq. (S22). All the above analyses, including symmetry identification and bulk transport property, are still valid for a randomly coupled lattice. Specifically, for symmetric lattices with $k_{s,1} = k_{s,2} = k_s$ and arbitrary *s*, Eq. (S22) can be simplified as $K_1(q) = K_{-1}(q) = 1 + 2\sum_{s=1}^{\beta} k_s \cos(sq)$ and $K_0(q) = 2 + 4\sum_{s=1}^{\beta} k_s$. In this case, the Hamiltonian is quasi-Hermitian, $\Gamma H = H^{\dagger}\Gamma$, with the positive-definite transform matrix Γ being expressed as

$$\Gamma = \begin{bmatrix} 2 + 4\sum_{s=1}^{\beta} k_s & -2 - 4\sum_{s=1}^{\beta} k_s \cos(sq) \\ -2 - 4\sum_{s=1}^{\beta} k_s \cos(sq) & 2 + 4\sum_{s=1}^{\beta} k_s \end{bmatrix} \tag{S23}$$

The spectrum of Γ is $\sigma(\Gamma) = 2 + 4\sum_{s=1}^{\beta} k_s \pm \left|2 + 4\sum_{s=1}^{\beta} k_s \cos(sq)\right| \geq 0$ (only if $q = 0$ that we have $\sigma(\Gamma) = 0$, which corresponds to the trivial translation mode with the two decay factors being $\lambda_1 = \lambda_2 = 1$). As a result, the decay spectrum is real in the whole BZ owing to the quasi-Hermitian character [18], and both TRS and IS are preserved with $T = \sigma_x$ and $P = I$. This implies that the static deformation response

actuated by a point load is always localized at the middle bulk region and forms a standing wave. Moreover, the null space of $H\left(q\right)$, or equivalently, the deformation blocked zero mode with $\lambda = 0$, is determined by $K_1\left(q\right) = 0$. Consequently, the density of zero modes can be enhanced by adding more long-range coupling terms, i.e., increasing β, which is of great advantages relevant to the deformation filter capability of the metamaterials [11]. Figure S3b shows the dispersion of $K_1\left(q\right)$ with identical $k_s = 1$ and different β. It can be seen that the number of zero modes (marked by the red dots) is equal to β. For this case, we have the following expression

$$K_1\left(q\right) = 1 + 2\sum\nolimits_{s=1}^{\beta} \cos\left(sq\right) = \frac{\sin\left(\beta + 1/2\right)q}{\sin q/2} \quad \text{(S24)}$$

which has β zero points within the half BZ. For asymmetric lattices (i.e., there is at least a pair of diagonal bars having $k_{s,1} \neq k_{s,2}$), TRS and IS are simultaneously broken and combined PTS is preserved, as the lattice is neither reflection symmetric about the *n* nor the *m* axes, but is still invariant under the 2D inversion.

VII. Generality of the transmission theory for static deformation fields

Our theory is general and applicable to a wide range of lattice materials with random crystallographic structures and coupling effects. In addition, since the sinusoidal patterns form a complete set of bases, any loading instance applied on the boundary can be decomposed and the associated bulk deformation field can be characterized by our theory. The only restriction is that the lattice is periodically extended so that the SR solution can be well defined on the scale of the unit cell. Below, we provide detailed analyses of the localization of static deformation fields in three additional lattices and isotropic continua given in Fig. 3. We also discuss potential applications of our theory to anisotropic media.

1. Modified X-braced lattice

First, consider a modified X-braced lattice with a single DoF and next-nearest-neighbor diagonal couplings (k_3 and k_4) between the nodes $\left(m,n\right)$ and $\left(m \pm 2, n \pm 1\right)$,

as schematically shown in Fig. 3a, which is far beyond the Maxwell's limit [19]. The added long-range couplings dramatically enrich the transmission property of the injected wave packet by carefully devising the decay spectrum in a large parameter space. Figure 3b depicts the colormap of $v_g(0)$ in the $k_3 - k_4$ space, where the nearest-neighbor couplings are fixed as $(k_1, k_2) = (1, 10)$. Evidently, the upper bound of the group velocity in X-braced lattices, i.e., $|v_g(0)| \le 1$, is compromised in the modified lattices due to the long-range coupling effects. Indeed, the maximum of $|v_g(0)|$ is now approaching 2, for the extreme limit when k_3 (or k_4) is dominant. As an illustration, the top panel of Fig. 3c shows the simulated displacement field under a point load applied on the top boundary in a modified lattice with $(k_3, k_4) = (1, 30)$ (marked by the white square in Fig. 3b). The excited deformation mode is guided towards a diagonal path with a large traveling angle pertaining to the vertical axis. The corresponding spatial evolution of the peak response $m_{\max}(n)$ is shown in the bottom panel of Fig. 3c, upon which the group velocity can be fitted as $\Delta m_{\max}/\Delta n = -1.51$, which is close to the theoretical prediction, $d\,\mathrm{Im}(\eta)/dq = -1.55$. Note that only the first few columns are selected in the calculation, since the bottom boundary effect is magnified in the deep bulk (e.g., when $n > 25$). In addition, there is a set of stiffness coefficients in the 2D parameter space such that $|v_g(0)| = 0$, corresponding to a localized wave packet, as marked by the dashed black line in Fig. 3b. These non-propagating modes arise from the destructive interference between the nearest- and next-nearest-neighbor couplings, even though the lattice has no TRS or IS. This is verified in Fig. 3d for $(k_3, k_4) = (16.95, 12.45)$ (marked by the green dot in Fig. 3b) where the injected wave packet is localized at the middle bulk region without a deflection, thereby forming a standing wave. The prediction of non-propagating modes by our transmission theory is even more subtle in modified X-braced lattices, as it is in general unable to identify a biased spatial direction where the deformation is accumulated (e.g., along k_1 or k_2 in X-braced lattices), when all four diagonal coupling stiffnesses are randomly set. Figure 3g depicts the simulated group velocities (blue dots) for these non-propagating modes (with k_3 acted as a variable), which are well constrained at the neighbor of $|v_g(0)| = 0$ (black line). Therefore, the

introduction of long-range couplings can largely enhance the capacity of controlling and guiding the static deformations in lattice materials.

2. Triangular lattice

Then, consider a triangular lattice shown in Fig. 3h (assuming only one DoF). The lattice vectors along the nonorthogonal *m* and *n* axes are $\boldsymbol{a}_1 = (1,0)$ and $\boldsymbol{a}_2 = (0.5,1)$, respectively (projected on the orthogonal basis $x - y$). The horizontal coupling stiffness is unitized while the diagonal stiffnesses are set as k_1 and k_2, see Fig. 3h. The real-space equilibrium equation for node (m,n) (with $n > 0$) is given by

$$k_1 u_{m,n-1} + k_1 u_{m,n+1} + k_2 u_{m+1,n-1} + k_2 u_{m-1,n+1} + u_{m+1,n} + u_{m-1,n} = k_t u_{m,n} \tag{S25}$$

with $k_t = 2(1 + k_1 + k_2)$. Inserting the SR solution to Eq. (S25) yields the decay spectrum. In particular, it is found that $v_g(q) = -0.5$ when $k_1 = k_2$ (Fig. 3i). This implies that the transfer of the wave packet along the *m* direction is given by $\Delta m = -0.5\Delta n$. After transforming it to the orthogonal basis, we arrive at a standing wave solution, $\Delta x = 0$ with arbitrary Δy, consistent with the spatially symmetric nature of a triangular lattice with $k_1 = k_2$ (see the simulated displacement field shown in Fig. 3j). Imposing $k_1 \neq k_2$ can induce unidirectional stress focusing at diagonal paths analogous to asymmetric X-braced lattices, albeit the group velocity derived from the imaginary spectrum should be distinguished from that measured on the orthogonal basis. This example demonstrates the feasibility of our theory for characterizing the transmission of static deformation fields in non-rectangular lattices.

3. Regular kagome lattice

As another paradigm, we focus on the collective deformation mode in a regular kagome lattice, as shown in Fig. 3k, which is comprised of an array of equilateral triangles. The unit cell contains three nodes with a total of six DoFs. The lattice vectors along the *m* and *n* axes are $\boldsymbol{a}_1 = (2,0)$ and $\boldsymbol{a}_2 = (1,\sqrt{3})$, respectively, and we impose a uniform coupling stiffness within the whole material. It is known that the kagome lattice is at the margin of mechanical instability and supports the zero-energy

deformation mode localized at the floppy edge, which is dictated by a topological polarization [20,21]. The bulk equilibrium equation can be gathered in matrix form and by employing the SR solution, $\boldsymbol{u}_{m,n} = \boldsymbol{p}e^{iqm}e^{-\eta n}$ with $\boldsymbol{p}$ the 6×1 polarization vector, it is solved that $e^{-\eta_1} = 1$ and $e^{-\eta_2} = e^{iq}$. Both solutions are zero-energy floppy deformation modes since they do not decay in the bulk (i.e., $\left|e^{-\eta}\right| = 1$). For example, the Bloch eigenvector of η_1 (projected on the orthogonal basis) is $\boldsymbol{p}_1 = \left(0,0,-\sqrt{3}/2,-1/2,0,-1\right)^T$, corresponding to a counterclockwise /clockwise rotation of all upward/downward triangles pivoted at node 1 and is known as the Guest-Huchinson mode [19], as schematically shown in Fig. 3l. For another branch η_2, we have $\boldsymbol{p}_2 = \left(0,-1,\sqrt{3}/2,-1/2,0,0\right)^T$. In specific, the collective deformation mode at $q = \pi$ is associated with the staggered rotations of adjacent upward (or downward) triangles along both the *m* and *n* axes, see Fig. 3m. The group velocities of these two branches are respectively $v_{g,1} = 0$ and $v_{g,2} = -1$, and are associated with the signal transmission towards the right and left boundaries, with an equal skewing rate pertaining to the vertical axis. A superposition of these polarizations yields a uniform translation, $\left|\boldsymbol{p}_{1+2}\right| = \left(0,1,0,1,0,1\right)^T$. Hence, for a point load applied on the top boundary along the vertical direction, the induced bulk deformation field will skew towards the left and right boundaries symmetrically, and this prediction is well corroborated by existing experiments [22].

4. Continuous elastic systems

Our theory is also applicable to continuous elastic systems once the decay spectrum is obtained. For example, the decay spectrum of an isotropic elasticity is $\eta = \pm q$, implying a zero group velocity of the incident wave packet and giving rise to a localized deformation mode in the middle bulk region. This conclusion is irrelevant to the constituent material's properties, as confirmed in Fig. 3n and o. This minimal example, despite being primary and can be explained by the elasticity theory, renders the first attempt for mapping the 2D continuous deformation field to the 1D wave motion. It also indicates that only anisotropic media can support asymmetric localization of bulk stress fields. Here, we draw our inspiration from an asymmetric

X-braced lattice which when the diagonal bars are densely enough, can be homogenized as a fiber-reinforced composite with unequal mechanical strengths of longitudinal and transverse fibers. Indeed, we have conducted numerical simulations by constructing a rectangular sample of plain-strain composites with their orthogonal fibers being 45° upward with respect to the horizontal surface. The result shows an asymmetric bifurcation of the bulk deformation field under an applied point load. This may serve as the foundation for tailoring the asymmetric stress localization in continuous elastic materials. However, many open questions remain as to extending our theory to anisotropic continua (e.g., the construction of band structure, the validation of symmetry analysis, and the synergies of building blocks).

VIII. Mechanical analogue of the non-Hermitian SSH model

This section provides an analysis of the mechanical analogue of the non-Hermitian Su-Schrieffer-Heeger (SSH) model illustrated in the main text (i.e., a diatomic rhombus lattice with asymmetric lattice configurations), including the derivation of the Bloch Hamiltonian, symmetry identification, band topology analysis, and the transmission of wave packets.

1 Bloch Hamiltonian

For the 2D compound rhombus lattice shown in Fig. 4a, where the PBC is applied along the *m* axis while the lattice extends infinitely along the *n* axis ($n \to \infty$), the nodal equilibrium equations of the unit cell (m,n) with $n > 0$ are given by

$$\begin{cases} k_1 u_{m,n-1}^{(2)} + k_3 u_{m-1,n-1}^{(2)} + k_2 u_{m,n+1}^{(2)} + k_4 u_{m-1,n+1}^{(2)} = k_{\mathrm{t}} u_{m,n}^{(1)} \\ k_2 u_{m,n-1}^{(1)} + k_4 u_{m+1,n-1}^{(1)} + k_1 u_{m,n+1}^{(1)} + k_3 u_{m+1,n+1}^{(1)} = k_{\mathrm{t}} u_{m,n}^{(2)} \end{cases} \tag{S26}$$

where $k_{\mathrm{t}} = k_1 + k_2 + k_3 + k_4$ is the total stiffness and $u_{m,n}^{(i)}$ denotes the vertical displacement of node P$_i$. The above equations can be rewritten as a vector summation, $\sum_{n'=-1}^{1} \sum_{m'=0}^{M-1} \boldsymbol{k}_{m-m' n-n'} \boldsymbol{u}_{m',n'} = 0$, where $\boldsymbol{u}_{m',n'}$ is the displacement vector with two DoFs per unit cell, $\boldsymbol{k}_{m-m' n-n'}$ is the stiffness matrix between (m',n') and (m,n) (see Section VI), and 2*M* is the site number along the *m* axis. The stiffness matrices are expressed as

$$\boldsymbol{k}_{11}=\begin{bmatrix}0 & k_3\\ 0 & 0\end{bmatrix}\quad \boldsymbol{k}_{01}=\begin{bmatrix}0 & k_1\\ k_2 & 0\end{bmatrix}\quad \boldsymbol{k}_{-11}=\begin{bmatrix}0 & 0\\ k_4 & 0\end{bmatrix}$$

$$\boldsymbol{k}_{1-1}=\begin{bmatrix}0 & k_4\\ 0 & 0\end{bmatrix}\quad \boldsymbol{k}_{0-1}=\begin{bmatrix}0 & k_2\\ k_1 & 0\end{bmatrix}\quad \boldsymbol{k}_{-1-1}=\begin{bmatrix}0 & 0\\ k_3 & 0\end{bmatrix}$$

$$\boldsymbol{k}_{10}=\begin{bmatrix}0 & 0\\ 0 & 0\end{bmatrix}\quad \boldsymbol{k}_{00}=\begin{bmatrix}-k_{\mathrm{t}} & 0\\ 0 & -k_{\mathrm{t}}\end{bmatrix}\quad \boldsymbol{k}_{-10}=\begin{bmatrix}0 & 0\\ 0 & 0\end{bmatrix} \tag{S27}$$

while the entries with indices larger than 1 are zero. Similar to Section VI where the periodical sequences of the stiffness matrices and displacement vectors are Fourier transformed [11], the above vector summation can be rearranged as an eigen-equation about the four bands Bloch Hamiltonian shown in the main text, i.e., Eq. (3). Note that the SR solution has been adopted to deduce the eigen-equation. When $k_1 = k_2$ and $k_3 = k_4$, both TRS and IS are preserved owing to the independent reflection symmetries along the two axes. The space inversion center is placed at the center of two adjacent nodes which, under the reflection operation, exchanges the two components within the polarization vector and therefore is selected as $P = I \otimes \sigma_x$. The time-reverse, on the other hand, only flips the decay factor ($\lambda \to \lambda^{-1}$) and has no effect on the polarization vector, for which it can be employed as $T = \sigma_x \otimes I$.

2 Reciprocal limit

Emulating a 1D diatomic mass-spring model with staggered coupling stiffnesses, Eq. (S26) can be reformulated in an effective interacting form at a given coordinate *m* via a space-attenuated solution, i.e., $\boldsymbol{u}_{m,n+1} = \lambda \boldsymbol{u}_{m,n}$

$$\begin{cases}\left(k_1\lambda^{-1}+k_2\lambda\right)\left(u_{m,0}^{(2)}-u_{m,0}^{(1)}\right)+\left(k_3\lambda^{-1}+k_4\lambda\right)\left(u_{m-1,0}^{(2)}-u_{m,0}^{(1)}\right)=\left(k_{\mathrm{t}}-k_1\lambda^{-1}-k_2\lambda-k_3\lambda^{-1}-k_4\lambda\right)u_{m,0}^{(1)}\\ \left(k_2\lambda^{-1}+k_1\lambda\right)\left(u_{m,0}^{(1)}-u_{m,0}^{(2)}\right)+\left(k_4\lambda^{-1}+k_3\lambda\right)\left(u_{m+1,0}^{(1)}-u_{m,0}^{(2)}\right)=\left(k_{\mathrm{t}}-k_2\lambda^{-1}-k_1\lambda-k_4\lambda^{-1}-k_3\lambda\right)u_{m,0}^{(2)}\end{cases}$$
(S28)

Analogous to the dynamic counterpart, the coefficients in the left-hand-sides of Eq. (S28) can be regarded as the effective couplings between the nearest neighbors along the *m* axis (the right-hand-sides provide additional on-site potentials and can be mimicked by adding the ground springs). For example, $k_1\lambda^{-1}+k_2\lambda$ ($k_4\lambda^{-1}+k_3\lambda$) and

$k_2\lambda^{-1}+k_1\lambda$ ($k_3\lambda^{-1}+k_4\lambda$) denote the backward and forward intra-cellular (inter-cellular) interactions between the adjacent nodes, respectively, for which the system is non-reciprocal in the dynamic sense when either $k_1\lambda^{-1}+k_2\lambda \neq k_2\lambda^{-1}+k_1\lambda$ or $k_3\lambda^{-1}+k_4\lambda \neq k_4\lambda^{-1}+k_3\lambda$ is satisfied. Note that the former inequalities exclude the uniform translation (i.e., $\lambda=1$) that is trivially reciprocal. Although we can add coefficients to Eq. (S28) to alter the effective couplings, the following identity is always satisfied when the couplings (both intra- and inter-cellular) are reciprocal

$$\frac{k_1\lambda^{-1}+k_2\lambda}{k_2\lambda^{-1}+k_1\lambda}=\frac{k_3\lambda^{-1}+k_4\lambda}{k_4\lambda^{-1}+k_3\lambda} \tag{S29}$$

from which we can solve that $k_1k_4=k_2k_3$. In this case, the asymmetric parts of the effective couplings offset and the lattice is net-reciprocal with entire real spectrum.

3 Spectral winding number about the zero decay factor

For non-Hermitian systems with a skin effect [7,23], the nonzero spectral winding number for a point-gap enclosed by the PBC loop on the complex energy plane is a unique characteristic. The reference energy of the point-gap is usually chosen as the origin when sublattice symmetry is preserved. In this case, it can be shown that $w_0=\frac{1}{2\pi i}\oint_{\mathrm{BZ}} d\ln\left[\det\left(H\right)\right]$ and

$$\det\left[H(z)\right]=\frac{k_1k_2+k_3k_4+k_1k_4z+k_2k_3z^{-1}}{k_1k_2+k_3k_4+k_2k_3z+k_1k_4z^{-1}}=\frac{f_1(z)}{f_2(z)} \tag{S30}$$

where $z=e^{iq}$. Consequently, we have $\ln\det\left[H(z)\right]=\ln f_1(z)-\ln f_2(z)$ and the winding number can be rewritten as

$$w_0=\frac{1}{2\pi i}\oint_{|z|=1}\frac{f_1'(z)}{f_1(z)}dz-\frac{1}{2\pi i}\oint_{|z|=1}\frac{f_2'(z)}{f_2(z)}dz=w_1-w_2 \tag{S31}$$

where $w_{1,2}$ respectively denote the geometrical windings of $f_{1,2}(z)$ with respect to the origin. The above integral can be solved using the argument theorem [24], unless $f_{1,2}(z)$ have zeros/poles on the integral path $|z|=1$, which will be considered later.

Right now, we assume that no singularity occurs. Within the integration path, $|z|<1$, $f_{1,2}(z)$ have a single pole at $z=0$. Moreover, the two (real) zero points of $f_1(z)$ are located at

$$z_{1/2}=\frac{-(k_1k_2+k_3k_4)\pm|k_1k_2-k_3k_4|}{2k_1k_4} \tag{S32}$$

Furthermore, we have $z_1=-k_3/k_1$ and $z_2=-k_2/k_4$ ($z_1=-k_2/k_4$ and $z_2=-k_3/k_1$) when $k_1k_2\geq k_3k_4$ ($k_1k_2<k_3k_4$). Similarly, the zero points of $f_2(z)$ are $\overline{z}_1=-k_4/k_2$ and $\overline{z}_2=-k_1/k_3$ ($\overline{z}_1=-k_1/k_3$ and $\overline{z}_2=-k_4/k_2$) for $k_1k_2\geq k_3k_4$ ($k_1k_2<k_3k_4$). The winding numbers $w_{1,2}$ (thereby w_0) depend on the difference between the number of zeros and poles of $f_{1,2}(z)$, and the result is summarized as follows:

1. $w_0=0$ when $k_1>k_3$ and $k_2>k_4$;
2. $w_0=0$ when $k_1<k_3$ and $k_2<k_4$;
3. $w_0=2$ when $k_1>k_3$ and $k_2<k_4$;
4. $w_0=-2$ when $k_1<k_3$ and $k_2>k_4$.

The inequalities are strictly satisfied under the above conditions; otherwise, the decay spectrum crosses the origin and the winding number is ill-defined. Note that the winding number is contributed from all four decay spectra, i.e., $\lambda_1-\lambda_4$. Now back to the earlier question. When $f_{1,2}(z)$ have zeros on the path $|z|=1$, it can be solved that $k_1=k_3$ and $k_2=k_4$. This corresponds to the reciprocal limit ($k_1k_4=k_2k_3$) obtained in Eq. (S29), in which the decay spectrum is real and the point-gap no longer survives. Hence, the above topological classification is unambiguous for any given stiffness parameters, and the change of w_0 signifies a topological phase transition. For example, the curved PBC spectrum transits from the unknotted state ($|w_0|=2$, Fig. 4b) to the gapless (ill-defined w_0, Fig. 4c) and unlinked states ($w_0=0$, Fig. 4d) [25] shown in the main text.

4 Non-Bloch band theory

A hallmark of non-Hermitian systems is the inconsistence between the PBC and OBC spectra along with the breakdown of bulk-edge correspondence (BEC) [26]. Particularly, the topological zero modes emerge even in the gapless phase with the two PBC bands touching each other, see Fig. 4c, and the conventional Bloch winding number defined on the BZ fails to predict the zero modes. This can be resolved with the emergent non-Bloch band theory [27] that adequately takes the skin effect into account for an open boundary system. Specifically, the OBC (bulk) spectrum of a non-Hermitian system is determined by the characteristic polynomial, $f(z,\lambda)=\det\left[H(z)-\lambda\right]=0$ with $z=e^{i(q+i\kappa)}$ being the non-Bloch wavenumber and λ the decay factor, respectively. The former equation yields two solutions of z as a function of λ, among which the OBC spectrum and the corresponding generalized Brillouin zone (GBZ) are determined by $|z_1(\lambda)|=|z_2(\lambda)|$ [27]. In the critical phase where the OBC bulk bands degenerate and the topological zero modes begin to emerge (disappear), we must have $|z_1(0)|=|z_2(0)|$, from which the restored phase boundary is determined as $k_1k_2=k_3k_4$ and is indeed different from that of the PBC band degeneracy, i.e., $k_1=k_3$ or $k_2=k_4$.

5 Wave packet transmission

Finally, we show the applicability of the static transmission theory in diatomic rhombus lattices. According to Section V, the group velocity can still be formulated as $v_g(q)=dm_{\max}/dn=d\,\mathrm{Im}(\eta)/dq$. The difference is that in compound lattices, there are multiple bands with distinct polarizations and the incident wave packet must comply with the polarization of its centered wavenumber. Also, the transfer of wave packet peaks (i.e., $\Delta m_{\max}$) is evaluated on the scale of unit cells, not lattice sites. First, consider $(k_1,k_2,k_3,k_4)=(10,1,1,6)$. The imaginary spectrum shown in Fig. S4a has two branches. One of them crossing the origin at $q=0$ corresponds to the uniform translation of the whole lattice with a polarization $\boldsymbol{p}_1=(1,1)^T$ (marked by blue), while another branch has a singularity at $q=0$ (marked by red), with its polarization

$\boldsymbol{p}_2 = (1,-1)^T$ corresponding to the relative translation of two nested sublattices. Both branches have a slope (i.e., the group velocity) of -0.38 at $q=0$ when considering the 2π period of $\mathrm{Im}(\eta)$. Applying a point load at the center of the top boundary, the associated bulk displacement fields are shown in Fig. S4b and c. It can be seen that a unidirectional stress localization is observed for both polarizations. The fitted group velocities are -0.35, consistent with the theoretical prediction.

Furthermore, according to Section VIII.2, the diatomic rhombus lattice is net-reciprocal when $k_1k_4 = k_2k_3$. In this case, the decay factor λ (where $\lambda = e^{-\eta}$) is entirely real even if the lattice has no symmetries (e.g., TRS or IS). As an illustration, we set $(k_1,k_2,k_3,k_4) = (10,2,5,1)$. The associated imaginary decay spectrum shown in Fig. S4d is 0 (for $\lambda > 0$) or π (for $\lambda < 0$) with a vanishing slope. The simulated bulk displacement fields are shown in Fig. S4e and f, where the incident wave packets are localized in the middle bulk region and behave as standing waves, consistent with their vanishing group velocities. Hence, we have shown the applicability of our theory even in compound lattices with multiple dispersion bands and polarizations.

IX. 3D static Rayleigh model

1. Continuum model

Consider a semi-infinite 3D isotropic elasticity with the boundary at the top surface, $l=0$. The equilibrium equations under the infinitesimal strain field are given by (without body force)

$$\begin{cases} \dfrac{1}{1-2\nu}\left(\dfrac{\partial^2 u_m}{\partial m^2} + \dfrac{\partial^2 u_n}{\partial m \partial n} + \dfrac{\partial^2 u_l}{\partial m \partial l}\right) + \nabla^2 u_m = 0 \\ \dfrac{1}{1-2\nu}\left(\dfrac{\partial^2 u_m}{\partial m \partial n} + \dfrac{\partial^2 u_n}{\partial n^2} + \dfrac{\partial^2 u_l}{\partial n \partial l}\right) + \nabla^2 u_n = 0 \\ \dfrac{1}{1-2\nu}\left(\dfrac{\partial^2 u_m}{\partial m \partial l} + \dfrac{\partial^2 u_n}{\partial n \partial l} + \dfrac{\partial^2 u_l}{\partial l^2}\right) + \nabla^2 u_l = 0 \end{cases} \tag{S33}$$

where $u_{m/n}(m,n,l)$ denotes the in-plane displacements while $u_l(m,n,l)$ denotes the out-of-plane displacement. The solution of the above boundary value problem

strongly depends on the prescribed boundary condition. Recall that the surface Rayleigh wave takes an exponential decay rate away from the free surface. Here, we take the surface SR mode (i.e., $u_l(m,n,l) = e^{i(q_m m + q_n n)} e^{-\eta l}$) as a trial solution of Eq. (S33) in view of its absent inertial force. Furthermore, the in-plane displacement components are supposed to be linearly proportional to u_l, i.e., $u_{m/n} = C_{1/2} u_l$, so that a collective deformation mode with all points sharing the same decay factor can be obtained. Indeed, this special solution tailors a delicate control of the applied boundary load and is hard to be implemented for a genuine continuum, but it indeed sheds light on the exploration of the SR mode in discretized lattice materials whose pin-joint structure provides a practically accessible loading method, as demonstrated in the next subsection. The problem is solvable because we have three variables (C_1, C_2 and η) and three independent equations in Eq. (S33). Inspired by the 2D Lame's equation where the decay factor takes a linear dispersion about the wavenumber, we may select a similar conic dispersion in the 3D case with a unitized coefficient, i.e., $\eta(\boldsymbol{q}) = \sqrt{q_m^2 + q_n^2}$, owing to the isotropic nature of the continuum that favors no directional bias among *mnl*. Note another negative branch with $\eta(\boldsymbol{q}) = -\sqrt{q_m^2 + q_n^2}$ is guaranteed by the TRS and will not be considered for the sake of brevity. Substituting these solutions into Eq. (S33), we obtain the following set of homogeneous equations

$$\begin{bmatrix} q_m^2 & q_m q_n & iq_m \\ q_m q_n & q_n^2 & iq_n \\ iq_m & iq_n & -1 \end{bmatrix} \begin{Bmatrix} C_1 \\ C_2 \\ \eta \end{Bmatrix} = 0 \tag{S34}$$

The coefficient matrix in Eq. (S34) should have a null determinant to ensure a nontrivial solution. In fact, from the third equation we have $\eta = iq_m C_1 + iq_n C_2$, which in turn trivializes the first two equations, i.e., they are self-balanced. In other words, it means that only one equation in Eq. (S34) is independent. As a result, by selecting a proper set of coefficients C_1 and C_2 that satisfies the third equation, the trial solution, i.e., the surface SR mode with a conic dispersion of the decay spectrum, is indeed an admissible solution of Eq. (S33). Along the principal axis where $C_2 = 0$, we have a canonical form $C_1 = -i\sqrt{q_m^2 + q_n^2} / q_m$. Finally, the physically acceptable displacements can be extracted from the real or imaginary part of the exponents. For example, we

have $u_m = -\eta \cos(q_m m + q_n n) e^{-\eta l} / q_m$, $u_n = 0$ and $u_l = \sin(q_m m + q_n n) e^{-\eta l}$ in the principal basis. One can check that the former solutions indeed satisfy Eq. (S33).

2. Lattice model

Next, we consider a practically implementable lattice model in terms of the surface SR solution. We take a single DoF case as an illustration. For a semi-infinite cubic lattice with normalized lattice constants and a free boundary at $l = 0$, the nodal position is labeled by three indices, (m, n, l), with *m* and *n* denoting the in-plane indices and *l* the layer index, respectively. The (asymmetric) diagonal interlayer stiffnesses between the nearest neighbors are k_1 and k_2 in the $m-l$ plane and k_3 and k_4 in the $n-l$ plane, respectively. The vertical interlayer stiffness is unitized by convention, while the intralayer coupling is neglected. Akin to the 2D lattice illustrated above, the nodal equilibrium equation can be arranged as

$$\sum_{l'=l-1}^{l+1} \sum_{m'} \sum_{n'} k_{m-m',n-n',l-l'} u_{m',n',l'} = 0 \tag{S35}$$

Because only the nearest-neighbor couplings are considered, the nonzero stiffness elements are respectively $k_{0,0,1} = k_{0,0,-1} = 1$, $k_{1,0,1} = k_{-1,0,-1} = k_1$, $k_{1,0,-1} = k_{-1,0,1} = k_2$, $k_{0,1,1} = k_{0,-1,-1} = k_3$, $k_{0,-1,1} = k_{0,1,-1} = k_4$ and $k_{0,0,0} = -2(1 + k_1 + k_2 + k_3 + k_4)$. Analogous to the non-Hermitian SSH model in Section VIII, Eq. (S35) denotes the convolution of two 2D periodic sequences and can be rearranged as an eigen-equation about the following Bloch Hamiltonian by 2D Fourier transform

$$H(\boldsymbol{q}) = \begin{bmatrix} 0 & 1 \\ -\dfrac{1 + k_1 e^{iq_m} + k_2 e^{-iq_m} + k_3 e^{iq_n} + k_4 e^{-iq_n}}{1 + k_1 e^{-iq_m} + k_2 e^{iq_m} + k_3 e^{-iq_n} + k_4 e^{iq_n}} & \dfrac{2(1 + k_1 + k_2 + k_3 + k_4)}{1 + k_1 e^{-iq_m} + k_2 e^{iq_m} + k_3 e^{-iq_n} + k_4 e^{iq_n}} \end{bmatrix} \tag{S36}$$

where the surface SR solution $u_{m,n,l} = e^{i(q_m m + q_n n)} e^{-\eta l}$ is used with the eigenvalue being the decay factor $\lambda = e^{-\eta}$. Similar to the 2D lattice model, the redefined TRS and IS can be extracted from the unitary symmetries of the Hamiltonian. In this case, TRS tailors the mirror symmetry along the layered *l* axis while IS accounts for the 2D inversion symmetry in the $m-n$ plane. In the high-symmetry configuration with

$k_1 = k_2$ and $k_3 = k_4$, both TRS and IS are preserved with $T = \sigma_x$ and $P = I$, otherwise they are simultaneously broken and the PTS is preserved. The non-reciprocal lattices with unequal k_1 and k_2 (k_3 and k_4) sustain the skin effect with all OBC bulk modes being squeezed towards the boundaries or corners [28], depending on the stiffness contrast between k_1 and k_2 (k_3 and k_4). Moreover, the group velocity is defined as the gradient of the imaginary decay spectrum in the momentum space, namely, $\boldsymbol{v}_g(\boldsymbol{q}) = \nabla_{\boldsymbol{q}} \mathrm{Im}(\eta)$, and a unidirectional wave packet transport can be tailored in the TRS broken region. The left panels of Fig. S5a–c depict the imaginary decay spectra with different sets of stiffness parameters, with the black arrows marking the gradient of the spectra. The results show that the orientation of the group velocity is closely related to the stiffness patterns upon which the chirality of non-reciprocity can be tuned. For example, the wave packet propagates towards the $+n$ ($-m$) axis in Fig. S5a (Fig. S5b) where only the $n-l$ ($m-l$) plane reciprocity is broken, whereas a corner accumulation is found in Fig. S5c with bidirectional non-reciprocity. Note that the discontinuity of the imaginary spectrum in Fig. S5c originates from the branch cut singularity of the phase factor (i.e., $\mathrm{Im}(\eta) = -\arg(\lambda)$), which is constrained within the range $(-\pi, \pi]$. These results are further verified by numerical simulations, where a 3D lattice with the size $M \times N \times L = 21 \times 21 \times 16$ is constructed. The bottom boundary ($l = 15$) is fully anchored and a point load is applied at the center of the top layer ($l = 0$). A unidirectional transport of the excited deformation mode during the *l*-directional spatial evolution is evident, as shown in the right panels of Fig. S5a–c, which is a nontrivial manifestation of the broken TRS in such a 3D layered material. Note that only a portion of layers far from the bottom boundary is presented here to eliminate the boundary effect. This directional transport facilitates a 3D stress guide with higher tunability compared with the 2D one. Meanwhile, these layered 3D lattice materials can be employed to emulate the 2D topological waves with targeted non-reciprocal couplings, which may facilitate the exploration of higher-order skin effects [29] and hybrid skin-topological corner modes [30]. A geometry dependent skin effect can also be tailored in a layer structure with irregular in-plane geometry and stacked along the *l* axis [31], which deserves further study.

X. Comparison of topological matters in synthetic dimensions

In this section, we give a brief comment about the proposed space-time mapping strategy in the SR model and the well-studied topological quantum dynamics in the synthetic dimensions. Modern manufacturing technologies allow us to explore sophisticated higher-dimensional topological phases of matter in an easily implemented system with reduced spatial dimensions, according to a synthesized parametric space called the synthetic dimension [32]. In this set up, one or more system parameters, including the frequency mode of a light cavity [33], the hopping amplitude between acoustic waveguides [34], and even the coupling stiffness of a mechanical mass-spring chain [35], can be extracted and reinterpreted to facilitate an additional DoF that acts as a compensation of the originally restricted physical spaces. Conversely, an extra space dimension in a higher-dimensional system can also be employed to study the lower-dimensional topological physics, such as the dimension enhanced photonic Floquet insulators where the inter-waveguide couplings along the propagating (z) direction are periodically modulated and a 2D quantum Hall effect can be mimicked [36], and the reduced Chern-insulator phase as a slice of the 3D Weyl semimetal without breaking TRS [37,38]. These two paradigms, which intrinsically concern dynamic wave motion, are very different from the SR model in this study where time is completely mapped out.

For a comparison of these different models, we focus on a purely static problem associated with the lattice deformation, as illustrated in Fig. S6a. This new set up brings out several subtle issues. For example, the time-related symmetries, such as TRS and PTS, are concretized as the crystalline symmetries, and the broken continuity along the time dimension provides a more intuitive scheme that breaks TRS (e.g., asymmetric arrangement of the diagonal couplings in X-braced lattices). By contrast, the discretization of time in wave dynamics is usually for mathematical intentions. Consider a 1D mass-spring chain whose wave dynamics is governed by $\ddot{\boldsymbol{u}}(t)+\Gamma\dot{\boldsymbol{u}}(t)+\boldsymbol{D}\boldsymbol{u}(t)=0$, where $\boldsymbol{u}$ is the displacement vector, $\boldsymbol{D}$ is the dynamic matrix and Γ is the velocity-related coupling matrix, such as viscous damping. The time derivative can be approximated by the finite difference method, as what has been done in Section IV for a spatial dimension. This is feasible since we can always find a narrow time interval among which the system evolves convergently under the

harmonic wave motion. In other words, it means that there is no singularity in particles' worldline. As a result, we obtain the following discretized wave equation in real space

$$\left(\boldsymbol{I}-2\Gamma\right)\boldsymbol{u}_{t-1}+\left(\boldsymbol{I}+2\Gamma\right)\boldsymbol{u}_{t+1}+\left(\boldsymbol{D}-2\boldsymbol{I}\right)\boldsymbol{u}_{t}=0 \tag{S37}$$

with $t\pm 1$ denoting the unit time increment, see Fig. S6b. Evidently, the nonzero Γ breaks TRS owing to the associated first-order derivative in the wave equation, or more visually, the asymmetric coupling effect between different time instances. On the other hand, the bulk equilibrium equation of a planar X-braced lattice is

$$\boldsymbol{K}_1\boldsymbol{u}_{n-1}+\boldsymbol{K}_2\boldsymbol{u}_{n+1}+\boldsymbol{K}_0\boldsymbol{u}_n=0 \tag{S38}$$

where $\boldsymbol{u}_n$ denotes the displacement vector for the *n*th row of the lattice and $\boldsymbol{K}_i$ is the coupling matrix between adjacent rows. We have $\boldsymbol{K}_1\neq\boldsymbol{K}_2$ when the diagonal stiffnesses are asymmetrically assigned (i.e., $k_1\neq k_2$). The correspondence between Eqs. (S37) and (S38) is clear, and the broken TRS can indeed be attributed to the asymmetric lattice configuration in our static model. It should be noted that Γ is usually a diagonal matrix and only couples a single oscillator itself at different time instances (Fig. S6b), whereas $\boldsymbol{K}_1$ and $\boldsymbol{K}_2$ are tridiagonal for which the spacetime reciprocities are simultaneously broken. The divided narrow time interval in the discretized Eq. (S37) indicates that the dynamic system is local in time. This, however, can be quite nontrivial in our model, since the isolated lattice array provides more paradigms for linking adjacent sites along both the space and quasi-time domains and hence, induces the non-locality of time and intriguing phenomena, such as the roton-like dispersion [39] of the decay spectrum and the modal inversion during the spatial evolution [11].

In this sense, our model provides an unprecedented venue for breaking time-translational symmetry and engineering the coupling effects along the evolution space, which is essentially different from the current studies in synthetic dimensions that concern dynamic evolution [32]. At the same time, it is also feasible to mimic a synthetic topological matter in our static model since the concretization of the quasi-time provides a direct designing strategy for analogous time-harmonic modulation in wave dynamics.

A prototypical example induced by such modulation is the topological pumping effect, where the lattice Hamiltonian in a 1D/2D/3D system is periodic with some parameters and the 2D/4D/6D quantum Hall effect can be effectively emulated [40–42]. These time-dependent modulations can be replaced by the *n*-directional spatial modulations in our SR model. Consider a compound square lattice with three nodes per unit cell, as shown in Fig. S6c. The nodes are restricted to translate along the *n* axis. The bar stiffnesses along the *n* axis are unitized (gray bars) and are taken as a function of *n* for the *m*-directionally aligned bars (blue, green and red bars), i.e., $k_i(n) = k\left[1 + k_d \cos(\omega n + \varphi_i)\right]$ with ω and φ_i the modulation frequency and the initial phase factor, respectively, and $i = 1,\ 2,\ 3$. The nodal equilibrium equations for the bulk unit cells (m,n) with $n > 0$ are given by

$$\begin{cases} u_{m,n-1}^{(1)} + u_{m,n+1}^{(1)} + k_1 u_{m,n}^{(2)} + k_3 u_{m-1,n}^{(3)} = k_\mathrm{t}^1 u_{m,n}^{(1)} \\ u_{m,n-1}^{(2)} + u_{m,n+1}^{(2)} + k_1 u_{m,n}^{(1)} + k_2 u_{m,n}^{(3)} = k_\mathrm{t}^2 u_{m,n}^{(2)} \\ u_{m,n-1}^{(3)} + u_{m,n+1}^{(3)} + k_2 u_{m,n}^{(2)} + k_3 u_{m+1,n}^{(1)} = k_\mathrm{t}^3 u_{m,n}^{(3)} \end{cases} \tag{S39}$$

where $u_{m,n}^{(i)}$ is the displacement of node P*i* (Fig. S6c). The total stiffnesses k_t^i are defined as

$$\begin{cases} k_\mathrm{t}^1 = 2 + k_1 + k_3 \\ k_\mathrm{t}^2 = 2 + k_1 + k_2 \\ k_\mathrm{t}^3 = 2 + k_2 + k_3 \end{cases} \tag{S40}$$

Applying the SR solution $\boldsymbol{u}_{m,n} = e^{iqm}\lambda^n \boldsymbol{p}$ with $\boldsymbol{p}$ the polarization vector to Eq. (S39) yields the following eigenvalue problem

$$\begin{bmatrix} k_\mathrm{t}^1 & -k_1 & -k_3 e^{-iq} \\ -k_1 & k_\mathrm{t}^2 & -k_2 \\ -k_3 e^{iq} & -k_2 & k_\mathrm{t}^3 \end{bmatrix} \begin{Bmatrix} p_1 \\ p_2 \\ p_3 \end{Bmatrix} = \mu \begin{Bmatrix} p_1 \\ p_2 \\ p_3 \end{Bmatrix} \tag{S41}$$

with $\mu = \lambda + \lambda^{-1}$ being the eigenvalue, which is a function of both the momentum *q* and the space *n*. The slow variation ($\omega \ll 1$) of the bar stiffnesses during the *n*-directional evolution breaks TRS and leads to a nontrivial phase accumulation that is responsible for the topological band gap [35,43], within which the chiral edge states

encircle the 2D synthetic real space spanned by m and μ. This mode switching has an origin similar to the 2D quantum Hall effect [40], where the topological phase of the decay spectrum in the synthesized momentum space spanned by $(q,n)\in[-\pi,\pi]\oplus[0,2\pi/\omega]$ is characterized by the (first) Chern number, which is defined as the integration of the Berry curvature in the BZ [44]. The Chern numbers are $C=1$ for the top and bottom bands and $C=-2$ for the middle band, respectively. Thus, both two band gaps are topologically nontrivial with nonzero gap Chern numbers $C_g=\pm1$, which are defined as the summation of the band Chern numbers below the gap. These nontrivial band gaps support chiral edge states for an OBC system according to the BEC, as shown in Fig. S6d for the projected band structure along the n axis, with two pairs of crossed edge states being visible (blue and green lines). The corresponding displacement profiles for the paired edge states in the upper band gap are shown in Fig. S6e for an evolution cycle.

These topological edge states, initially localized at the left/right edge, are pumped to the opposite edge after a period of adiabatic evolution. The chirality of the edge states can be illustrated in the synthetic real space. The edge state migrates from the right (left) boundary to the left (right) boundary and then decreases (increases) its decay factor. Consequently, it can be mapped as a traveling wave packet with a counterclockwise trajectory along the edge of the 2D sample lying on the $m-\mu$ plane [45]. Combining the two edge states together results in an intact trajectory, which means that we need a double cycle to go back to the starting point, as pictorially shown in Fig. S6f. This periodicity doubling character is phenomenologically analogous to the Floquet time crystal with spontaneous broken of the discrete time-translational symmetry [46]. The chirality of the edge states can be flipped by reversing the modulation frequency, accompanied by the reversing of the associated Chern numbers of the decay spectrum and the band gap.

The above model is a nontrivial manifestation of the topological pumping effect in our static system, where the space n is naturally selected as the synthetic dimension along which the stiffness parameters are tuned and the edge states are adiabatically transferred. One thing that needs to be concerned is the confliction between the adiabatic theorem and Saint-Venant's principle. The former requires a sufficiently slow modulation of the system parameters compared with the internal evolution cycle

so that the Hamiltonian lies in its instantaneous eigenstate at every instant [45], which, in our model, tailors a large number of evolution cycles along the n axis. While Saint-Venant's principle argues that the bulk response induced by a boundary load is always localized and attenuates rapidly away from the edge. As a result, it is generally difficult to observe a static pumping effect in experiments as the lattice deformation is too weak after a large number of cycles. This might be resolved by considering the adiabatic evolution of a non-attenuated SR mode where $\mathrm{Re}(\eta)=0$ is maintained even with a varying parameter, such as the floppy deformation mode in isostatic lattices.

XI. Hunting the low-energy physics in static Rayleigh model

All the above illustrated models focus on the SR mode with a nonvanishing decay factor, i.e., $\eta \neq 0$, which is dubbed as the finite-energy mode in view of the correspondence between the decay factor and the energy/frequency in wave dynamics [47]. Note that the deformation blocked zero mode with $\lambda = 0$ is also a finite-energy mode (i.e., $\eta \to \infty$). A hallmark of the mechanical system is the intrinsic PHS, which states that the positive and negative frequency-wavenumber doublets are always pairwise (i.e., $\omega(-q) = -\omega(q)$ in Hermitian systems) and cannot be removed even when TRS is broken [4]. This compensates another branch of topological mechanics that focuses on the low-energy ($\omega \to 0$) static deformation mode in a patterned lattice material or continuum, such as the zero energy floppy mode and the state of self-stress in Maxwell lattices at the margin of mechanical stability [17,48]. On the other hand, the intrinsic PTS in the SR model specifies that $\eta(-q) = -\eta(q)$ and is analogous to the PHS. This naturally motivates us to explore the "low-decay factor" physics with $\eta \to 0$, following directly from the dynamic counterpart.

At first glance, this low-energy mode might seem trivial since it actually corresponds to the uniform translation of the entire lattice without any distortion. However, in the case of a zero frequency floppy mode in an isostatic lattice, such as the simplest 1D Kane-Lubensky chain [17], there is a set of nodal displacements where the bar extension/compression is zero. This is also true in our static model where the applied boundary load (i.e., a prescribed set of nodal displacements at $n = 0$) will not decay ($\eta = 0$) if no strain energy is stored in the bar elements, namely, the deformation is

floppy. In other words, when dealing with low-energy physics, either from frequency-based dynamics or decay factor-based statics, the two approaches should be strictly equivalent, not only in mathematics but also in physics. This offers a new perspective for exploring low-frequency vibration modes that are based on the SR model.

For 1D systems, let's consider a periodic mass-spring chain with random sublattice structures and coupling effects, as schematically shown in Fig. S7a. The system is passive without any velocity-related terms. The dynamic equations can be expressed as $\omega^2 u_{\alpha,i} = \sum_{\alpha',j} K_{\alpha i,\alpha' j} u_{\alpha',j}$ by applying the time-harmonic solution (the mass density is set to be unit), where *i* and α label the lattice site and internal DoF, respectively, and the summation is taken over the coupling range. These equations can be gathered as $\omega^2 \boldsymbol{u} = \boldsymbol{K}\boldsymbol{u}$ by incorporating all DoFs into a vector $\boldsymbol{u}$. Then, we aim to find a planar lattice such that the low-frequency vibration mode in the mass-spring chain can be strictly deduced from the 2D SR solution in such a lattice. To this end, we consider a paradigmatic square lattice, which is constructed through a direct stack of the mass-spring chain along the *n* axis with only nearest interchain couplings between $u_{\alpha,i}^{n\pm1}$ and $u_{\alpha,i}^{n}$ (i.e., no diagonal couplings, see Fig. S7b). The equilibrium equations are $\boldsymbol{u}_{n-1} + \boldsymbol{u}_{n+1} = \boldsymbol{K}'\boldsymbol{u}_n$ (the interchain coupling is unitized), which can be simplified as $\left(\lambda + \lambda^{-1}\right)\boldsymbol{u} = \boldsymbol{K}'\boldsymbol{u}$ by applying the space-attenuated solution, $\boldsymbol{u}_n = \lambda^n \boldsymbol{u}_0$ with $\boldsymbol{u}_0 = \boldsymbol{u}$ being the applied boundary load. The coupling matrix is $\boldsymbol{K}' = \boldsymbol{K} + 2\boldsymbol{I}$ with $\boldsymbol{I}$ the identity matrix owing to the acoustic sum rule [49]. Consequently, we have $\left(\lambda + \lambda^{-1} - 2\right)\boldsymbol{u} = \boldsymbol{K}\boldsymbol{u} \approx \eta^2 \boldsymbol{u}$ for a small η, and this approximation is strictly satisfied when $\eta \to 0$. This means that the low-frequency mode in any mass-spring chain, instead of being solved from the dynamic equations, can be deduced from an enlarged square lattice in terms of the SR mode.

The above analyses can be extended to 3D cases in a straightforward manner, where the 2D vibration system is stacked along the perpendicular (*l*) axis with only nearest couplings between adjacent layers, and the 2D vibration mode in the low-frequency range can be derived from the associated 3D SR mode in the layered cubic lattice. Figure S7c and d shows the vibration spectra and the corresponding decay spectra for various models in multi-dimensions, where a precise accordance between the two in

the low-energy range at $q \to 0$ can be observed, in addition to the left panel of Fig. S7c where the added ground springs shift the ground state away from the zero-energy. On the contrary, adding diagonal couplings between the stacked chains (layers) breaks the locality along the quasi-time and such a low-energy correspondence no longer validates. Nevertheless, these diagonal couplings do provide another route for exploring the anomalous finite-energy physics, such as the skin effect and the unidirectional stress localization explored in this study.

XII. Numerical simulations

All numerical simulations for lattice systems, including the measurement of group velocities and nodal displacements, are performed using the commercially available finite-element-method (FEM) software Ansys. To determine the group velocity, we construct a truss-like X-braced lattice (i.e., stretch dominant) with the size $M \times N = 101 \times 101$. A concentrated point load is applied at the center of one boundary, while the other boundary is fully anchored to eliminate trivial translation. The lattice nodes are restricted to translate either vertically (single DoF case) or both horizontally and vertically (double DoFs case). The simulated lattice deformations shown in Fig. 1d–g, and Fig. 3c and d are partially extracted from the vicinity of the loading point of the whole structure for better visualization. The group velocity of the wave packet deformation mode, defined as the skewing rate of its peak response during the spatial evolution, is measured from the averaged ratio between the migration of the displacement maximum ($\Delta m_{\max}$) and the evolution cycle (Δn), i.e., $v_g = \Delta m_{\max} / \Delta n$ with a sufficiently large cycle, $\Delta n \gg 1$, and the result is shown in Fig. 1c and Fig. 3g. Similar procedures are applied to the compound rhombus lattices with the size 51×51 (unit cells), with $\Delta m_{\max}$ evaluated on the scale of unit cells.

Four single DoF X-braced lattices, separated by domain walls, are assembled to form the heterostructure in Fig. 2a. Each part has a size of $M \times N = 8 \times 8$. The top and bottom boundaries are fixed while the left and right boundaries are traction-free. A point load along the n direction is applied at the junction (marked by the red dot) and the displacement fields within the whole structure are measured, as shown in Fig. 2b–h. In particular, there are randomly distributed ground springs connected to the lattice sites in Fig. 2h to verify the robustness of the directional transmission. The ground

springs behave as the effective on-site potentials and correspond to the Anderson-type disorders. In Fig. 2i, a single DoF X-braced lattice with irregular boundary geometry is constructed. A point load is applied on the upper left boundary while the incompletely coordinated nodes at the right boundary are fixed. The measured displacement field shows the unidirectional transmission of the boundary load and demonstrates the geometry irrelevance of the stress guide, since it is a bulk property. In Fig. 2 j–l, a heterostructure composed of left and right two parts (with single DoF) is constructed. A set of point loads applied on three specified nodes at the top boundary of the left part excite a wave packet that moves unidirectionally towards the interface, owing to the positive group velocity. A portion of lattice nodes at the interface is fixed to supply a proper boundary constraint for the right part. The displacement intensity is renormalized for the right part for better visualization.

To identify the symmetries in terms of the TZM, we construct a 12×3 lattice sample with the two side-most lattice nodes of the middle row (i.e., $m=0$ and $m=11$ with $n=0$) fully constrained as the fixed boundary condition. All nodes are restricted to translate vertically (along the n axis). The external load that corresponds to the eigenstate of the TZM is applied on the middle row, and the associated nodal displacements at $n=\pm1$ are measured to identify whether the symmetries are broken or preserved. The simulation results are shown in the top panels of Fig. 5d and e.

The simulations of isotropic continua shown in Fig. 3n–q are conducted using the FEM software Abaqus. A plane-strain rectangular sample with an aspect ratio $m_0=2n_0$ is constructed. A point load is applied at the middle of the top boundary while the bottom boundary is fixed. The meshes are squares with a total of 20,301 nodes. The peak locations of the wave packet in the bottom panels of Fig. 3n and o are extracted from the displacement fields of every set of horizontal nodes on the square mesh. Then, we Fourier transform these displacement fields and show their intensities in Fig. 3p as a function of n. The linear and exponential estimations of the wave packet attenuation in Fig. 3q correspond to a uniform translation and a SR solution with $\eta=q=\pi$ exerted on $n=0$, respectively, which are formulated as $A(n)=1-n/100$ (101 nodes along the n direction) and $A(n)=\exp(-\pi n)$, with A denoting the amplitude of the wave packet.

XIII. Experimental implementations

The frame-like lattice sample shown in Fig. 1a is 3D-printed by the thermoplastic polyurethane with a size of $M \times N = 13 \times 7$. The nodes are hexagonal prisms with a length of about 6.5cm to prevent them from out-of-plane buckling, as well as to avoid the intersections of diagonal bars. The horizontal coupling bars are discarded since they do not affect the bulk transmission [15], while the vertical and diagonal linkages are specially designed beams with thickened mid sections to enhance their compressibility and reduce buckling. The coupling strengths of diagonal bars are adjusted by changing the diameters and numbers of connected beams. The bottom of the frame is fixed on the baseboard (acrylic plate) and the left/right boundary is traction-free. A displacement-controlled compressive load is applied to the center of the top boundary with a magnitude of 6cm. For Fig. 1d and e, there are vertical sliders crossing every column of nodes (with a common coordinate m) to restrict redundant DoF components. Note that our system is quasistatic and immune to the material damping effect, as we focus on the stable deformation state for a long duration after loading. The in-plane nodal displacement fields are measured by the digital-image-correlation (DIC) method. Although these samples are monolithic and deviate from the pin-joint-based theoretical models, the measured results shown in Fig. 1d–g are well consistent with that simulated from the truss-like X-braced lattices. This provides added confidence in the generality of our theory.

The monolithic frame-like lattices in the right panels of Fig. 2b–g are also 3D-printed, with the size $M \times N = 17 \times 17$. The nodes are replaced by pillars and are connected by cylindrical beams (with a larger stiffness) or curved strings (with a smaller stiffness). Each lattice is composed of four subparts, as guided by Fig. 2b–g, and the coupling stiffnesses in each subpart are adjusted independently. Figure 8 provides photographs of these specimens, where the insets show the direction of cylindrical beams in each subpart. The top and bottom boundaries of the lattice are fixed on the baseboards, and a displacement load of about 12mm is applied at the junction of four parts (aligned along the $+n$ axis). The measured nodal displacement fields qualitatively agree with the simulation results, confirming the capability of tailoring ordered deformation modes in heterostructures.

Another experimental platform for detecting the non-Hermitian TZM is an assembled truss-like lattice with the same size as that in the simulation, see Fig. 5a. The lattice nodes, which are replaced by cylindrical pillars and are connected by the *n*-directionally aligned sliders that lie on the wing of the platform, can only translate along the *n* axis. The loads are applied through the match between the bottom ends of pillars and the baseboard. The couplings between adjacent pillars are provided by the linear springs embedded within the bars. The analogous Rayleigh-Ritz method proposed in previous studies [15,50] is employed here to stimulate a SR deformation mode, namely, applying an appropriate boundary condition so that all nodes share the same decay factor. The key difference is that the external load is now applied on the central row (i.e., $n=0$) of the 12×3 lattice sample (the side-most nodes are fixed as the boundary constraints, and there are five unit cells for each row, as illustrated in Fig. S9a) so that an original state at $n=1$ and a time-reversed state at $n=-1$ can be simultaneously excited. This allows us to judge whether the corresponding symmetries (i.e., TRS and PTS) are broken or preserved by checking the nodal displacements at $n=\pm1$, which are measured by the DIC method. If TRS is preserved, the original state and the time-reversed state share the same eigenmode including the TZM. This means that the applied load on $n=0$ can induce a deformation shielding at both $n=\pm1$ owing to the (nearing) zero decay factor ($\lambda=0$) of the TZM. Note that the zero mode only entails the force balance between the middle and adjacent one rows, i.e., between $n=0$ and $n=\pm1$, so that a 12×3 lattice sample suffices to observe the TZM. However, the experimental results shown in Fig. 5b–e indicate that only one of the two rows ($n=1$ or -1) enjoys a deformation shielding while a strong response is observed for another, a strong evidence of the broken TRS stemmed from the asymmetric lattice couplings. The PTS, on the other hand, requires that the eigenmodes (including the TZM) of the original state and the time-reversed state of the same structure share the reciprocal decay factors (λ and λ^{-1}), as well as oppositely biased spatial localizations (left and right boundaries) due to the skin effect. This is clearly presented in our experiment where only $n=1$ ($n=-1$) is deformation-free when a TZM that is localized at $m=1$ ($m=10$) is applied on $n=0$, see Fig. 5b–e for the deformation plots and measured responses.

Although we assume that the stiffnesses of the diagonal bars are constant in the current experiment, it is meaningful to explore the nonlinear effects on the stiffness.

Recall that the diagonal bar stiffness of the rhombus lattice is defined as the *n*-directional reaction force of the linking bar induced by the unit *n*-directional relative translation of two connecting nodes. For the linear assumption, the effective bar stiffness can be formulated as $k_{\mathrm{eff}}^{j}=k_{\mathrm{spring}}^{j}\cos^{2}\theta$, where k_{spring}^{j} is the spring constant with $j=1-4$, and $\theta=27.9^{\circ}$ is the relative angle between the diagonal bars and the *n* axis in the undeformed state. Hence, the linear reaction force is $F_{\mathrm{linear}}^{j}=k_{\mathrm{eff}}^{j}\Delta u$, where Δu is the (*n*-directional) relative translation of two nodes connecting the spring k_{spring}^{j}.

By contrast, the true reaction force is $F_{\mathrm{nonlinear}}^{j}=k_{\mathrm{spring}}^{j}\Delta l\cos\theta^{'}\Delta u$, where Δl is the elongation of the spring and $\theta^{'}$ is the transient relative angle after deformation. We have $\Delta l=\sqrt{n_0^2+m_0^2}-\sqrt{\left(n_0-\Delta u\right)^2+m_0^2}$ and $\cos\theta^{'}=\left(n_0-\Delta u\right)\big/\sqrt{\left(n_0-\Delta u\right)^2+m_0^2}$, where $n_0=99.5\mathrm{mm}$ and $m_0=52.6\mathrm{mm}$ are lattice constants along the *n* and *m* axes. In this experiment, the spring constants are selected as 0.2N/mm, 0.2N/mm, 0.3N/mm, 0.6N/mm for k_{spring}^{1} to k_{spring}^{4}, respectively, and the displacement load applied at $n=0$ corresponds to the eigenstate of the topological zero mode (dubbed as $\boldsymbol{\psi}$), with a maximum at $\boldsymbol{\psi}(1)$ being 24mm. As an illustration, Fig. S9b depicts the linear and nonlinear force-displacement curves of bar k_1, where Δu is shown in the range between -18.41mm and 24.75 mm, corresponding to the smallest and largest relative displacements measured in the experiment. Then, we numerically solve the nodal displacement fields at $n=\pm1$ by imposing the force balance condition of these nodes. For example, the force balance of the first node at $n=1$ requires that $F_{\mathrm{nonlinear}}^{3}\big|_{\Delta u=\boldsymbol{\psi}(0)-u_{1,1}}+F_{\mathrm{nonlinear}}^{1}\big|_{\Delta u=\boldsymbol{\psi}(2)-u_{1,1}}=0$, where $\boldsymbol{\psi}(0)=\boldsymbol{\psi}(11)=0$ denote fixed boundary conditions and $u_{1,1}$ is the displacement of that node, see Fig. S9a.

Figure S9c shows the calculated linear and nonlinear nodal displacement responses. The two solutions agree well with each other with minor deviations nearing the left boundary. Especially, under the applied zero mode (i.e., $\lambda=0$) at $n=0$ (black solid), a deformation shielding at $n=1$ (blue dashed) and a strong response at $n=-1$ (red dashed) are still observed even in the nonlinear scenery, which is a clear evidence of

the broken TRS. Similar analysis can be applied to another topological mode with $\lambda = \infty$. Hence, we can conclude that the nonlinear effect is minor for the symmetry identification and can be neglected in the current experiment.

The paired TZMs in the dimerized atomic chain have degenerate eigenvalues and a chiral conversion character [51]. They originate from the symmetric or anti-symmetric hybridization of two unidirectionally localized zero modes of a semi-infinite system. One of the zero modes has a deformation fully constrained at the sublattices P_1 (applied on $n = 0$), which in turn induces a response merely concentrated on the sublattices P_2 at $n = \pm 1$, owing to the special geometry of the rhombus lattice (Fig. 4a). Another zero mode is a mirror image of the former one and has an opposite effect, i.e., the deformation is constrained at P_2 ($n = 0$) and induces a nonzero response at P_1, $n = \pm 1$. This conclusion holds even in non-Hermitian systems with skin effect [52]. Thus, reversing the sign of either constituent zero mode (which converts the hybridization from symmetric to anti-symmetric) has no effect on the deformation intensities at $n = \pm 1$ that are defined as their absolute values. In other words, it means that the two TZMs have an equal impact on the deformation response. We have conducted an experiment with the applied loads at $n = 0$ containing both two zero modes (denoted as Mode1 and Mode2 in Fig. 5b and c) and the measured response is defined as their average value, as marked by the error bars in the bottom panels of Fig. 5d and e.

References

1. Hatano N, Nelson DR. Localization transitions in non-Hermitian quantum mechanics. *Phys Rev Lett* 1996;**77**:570–3.

2. Hatano N, Nelson DR. Vortex pinning and non-Hermitian quantum mechanics. *Phys Rev B* 1997;**56**:8651–73.

3. Ashida Y, Gong Z, Ueda M. Non-Hermitian physics. *Adv Phys* 2020;**69**:249–435.

4. Süsstrunk R, Huber SD. Classification of topological phonons in linear mechanical metamaterials. *Proc Natl Acad Sci* 2016;**113**:E4767.

5. Longhi S. Non-Hermitian skin effect beyond the tight-binding models. *Phys Rev B* 2021;**104**:125109.

6. Ge Z-Y, Zhang Y-R, Liu T *et al.* Topological band theory for non-Hermitian systems from the Dirac equation. *Phys Rev B* 2019;**100**:054105.

7. Kawabata K, Shiozaki K, Ueda M *et al.* Symmetry and topology in non-Hermitian physics. *Phys Rev X* 2019;**9**:041015.

8. Wang Y, Yousefzadeh B, Chen H *et al.* Observation of nonreciprocal wave propagation in a dynamic phononic lattice. *Phys Rev Lett* 2018;**121**:194301.

9. Chiu C-K, Teo JCY, Schnyder AP *et al.* Classification of topological quantum matter with symmetries. *Rev Mod Phys* 2016;**88**:035005.

10. Fruchart M, Zhou Y, Vitelli V. Dualities and non-Abelian mechanics. *Nature* 2020;**577**:636–40.

11. Karpov EG. Structural metamaterials with Saint-Venant edge effect reversal. *Acta Mater* 2017;**123**:245–54.

12. Hussein MI, Leamy MJ, Ruzzene M. Dynamics of phononic materials and structures: Historical origins, recent progress, and future outlook. *Appl Mech Rev* 2014;**66**, DOI: 10.1115/1.4026911.

13. Ibach H, Lüth H. *Solid-State Physics: An Introduction to Principles of Materials Science*. Berlin, Heidelberg: Springer Berlin Heidelberg, 2009.

14. Klein JT, Karpov EG. Exact analytical solutions in two dimensional plate-like mechanical metamaterials: State of free deformation in a topological cylinder. *Int J Mech Sci* 2020;**167**:105292.

15. Wang A, Meng Z, Chen CQ. Non-Hermitian topology in static mechanical metamaterials. *Sci Adv* 2023;**9**:eadf7299.

16. Stephen NG. Transfer matrix analysis of the elastostatics of one-dimensional repetitive structures. *Proc R Soc A* 2006:2245–70.

17. Kane CL, Lubensky TC. Topological boundary modes in isostatic lattices. *Nat Phys* 2014;**10**:39–45.

18. Scholtz FG, Geyer HB, Hahne FJW. Quasi-Hermitian operators in quantum mechanics and the variational principle. *Ann Phys* 1992;**213**:74–101.

19. Rocklin DZ, Zhou S, Sun K *et al.* Transformable topological mechanical metamaterials. *Nat Commun* 2017;**8**:14201.

20. Xiu H, Liu H, Poli A *et al.* Topological transformability and reprogrammability of multistable mechanical metamaterials. *Proc Natl Acad Sci* 2022;**119**:e2211725119.

21. Zhou Y, Zhang Y, Chen CQ. Amplitude-dependent boundary modes in topological mechanical lattices. *J Mech Phys Solids* 2021;**153**:104482.

22. Pishvar M, Harne RL. Soft topological metamaterials with pronounced polar elasticity in mechanical and dynamic behaviors. *Phys Rev Appl* 2020;**14**:044034.

23. Okuma N, Kawabata K, Shiozaki K *et al.* Topological origin of non-Hermitian skin effects. *Phys Rev Lett* 2020;**124**:086801.

24. Gong Z, Ashida Y, Kawabata K *et al.* Topological phases of non-Hermitian systems. *Phys Rev X* 2018;**8**:031079.

25. Hu H, Zhao E. Knots and non-Hermitian Bloch bands. *Phys Rev Lett* 2021;**126**:010401.

26. Yao S, Wang Z. Edge states and topological invariants of non-Hermitian systems. *Phys Rev Lett* 2018;**121**:086803.

27. Yokomizo K, Murakami S. Non-Bloch band theory of non-Hermitian systems. *Phys Rev Lett* 2019;**123**:066404.

28. Zhang X, Tian Y, Jiang J-H *et al.* Observation of higher-order non-Hermitian skin effect. *Nat Commun* 2021;**12**:5377.

29. Kawabata K, Sato M, Shiozaki K. Higher-order non-Hermitian skin effect. *Phys Rev B* 2020;**102**:205118.

30. Lee CH, Li L, Gong J. Hybrid higher-order skin-topological modes in nonreciprocal systems. *Phys Rev Lett* 2019;**123**:016805.

31. Zhang K, Yang Z, Fang C. Universal non-Hermitian skin effect in two and higher dimensions. *Nat Commun* 2022;**13**:2496.

32. Ozawa T, Price HM. Topological quantum matter in synthetic dimensions. *Nat Rev Phys* 2019;**1**:349–57.

33. Song Y, Liu W, Zheng L *et al.* Two-dimensional non-Hermitian skin effect in a synthetic photonic lattice. *Phys Rev Appl* 2020;**14**:064076.

34. Chen H, Zhang H, Wu Q *et al.* Creating synthetic spaces for higher-order topological sound transport. *Nat Commun* 2021;**12**:5028.

35. Rosa MIN, Pal RK, Arruda JRF *et al.* Edge states and topological pumping in spatially modulated elastic lattices. *Phys Rev Lett* 2019;**123**:034301.

36. Rechtsman MC, Zeuner JM, Plotnik Y *et al.* Photonic Floquet topological insulators. *Nature* 2013;**496**:196–200.

37. Xiao M, Chen W-J, He W-Y *et al.* Synthetic gauge flux and Weyl points in acoustic systems. *Nat Phys* 2015;**11**:920–4.

38. Li F, Huang X, Lu J *et al.* Weyl points and Fermi arcs in a chiral phononic crystal. *Nat Phys* 2018;**14**:30–4.

39. Chen Y, Kadic M, Wegener M. Roton-like acoustical dispersion relations in 3D metamaterials. *Nat Commun* 2021;**12**:3278.

40. Kraus YE, Lahini Y, Ringel Z *et al.* Topological states and adiabatic pumping in quasicrystals. *Phys Rev Lett* 2012;**109**:106402.

41. Zilberberg O, Huang S, Guglielmon J *et al.* Photonic topological boundary pumping as a probe of 4D quantum Hall physics. *Nature* 2018;**553**:59–62.

42. Petrides I, Price HM, Zilberberg O. Six-dimensional quantum Hall effect and three-dimensional topological pumps. *Phys Rev B* 2018;**98**:125431.

43. Nakajima S, Tomita T, Taie S *et al.* Topological Thouless pumping of ultracold fermions. *Nat Phys* 2016;**12**:296–300.

44. Liao Y, Zhou X. Topological pumping in doubly modulated mechanical systems. *Phys Rev Appl* 2022;**17**:034076.

45. Nassar H, Chen H, Norris AN *et al.* Quantization of band tilting in modulated phononic crystals. *Phys Rev B* 2018;**97**:014305.

46. Else DV, Bauer B, Nayak C. Floquet time crystals. *Phys Rev Lett* 2016;**117**:090402.

47. Huber SD. Topological mechanics. *Nat Phys* 2016;**12**:621–3.

48. Sun K, Mao X. Continuum theory for topological edge soft modes. *Phys Rev Lett* 2020;**124**:207601.

49. Ding Z-K, Zeng Y-J, Pan H *et al.* Edge states of topological acoustic phonons in graphene zigzag nanoribbons. *Phys Rev B* 2022;**106**:L121401.

50. Wang A, Zhou Y, Chen CQ. Topological mechanics beyond wave dynamics. *J Mech Phys Solids* 2023;**173**:105197.

51. Tzortzakakis AF, Katsaris A, Palaiodimopoulos NE *et al.* Topological edge states of the PT-symmetric Su-Schrieffer-Heeger model: An effective two-state description. *Phys Rev A* 2022;**106**:023513.

52. Zhu W, Teo WX, Li L *et al.* Delocalization of topological edge states. *Phys Rev B* 2021;**103**:195414.

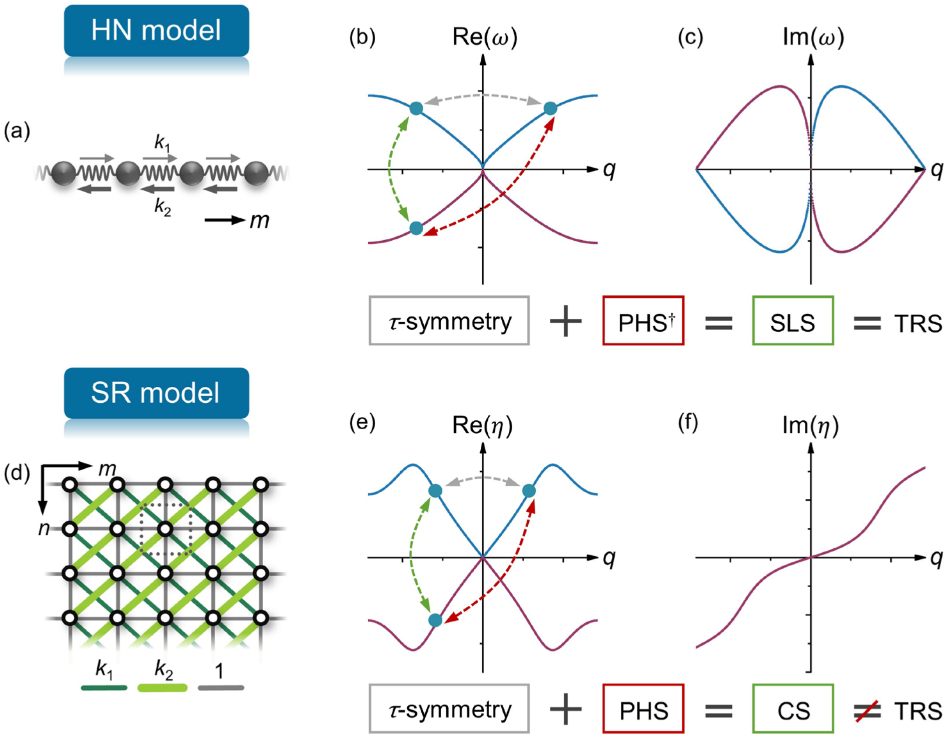

Figure S1. The underlying symmetries in the mechanical HN model and SR model. (a) Schematic of a mechanical HN model. (b and c) Corresponding real (b) and imaginary (c) parts of the vibration spectrum. The mass density is $\mu = 1$, while the stiffnesses are $k_1 = 3$ and $k_2 = 1$. (d) Schematic of an X-braced lattice with 1 DoF. (e and f) Corresponding real (e) and imaginary (f) parts of the decay spectrum. The stiffnesses are $k_1 = 3$ and $k_2 = 1$.

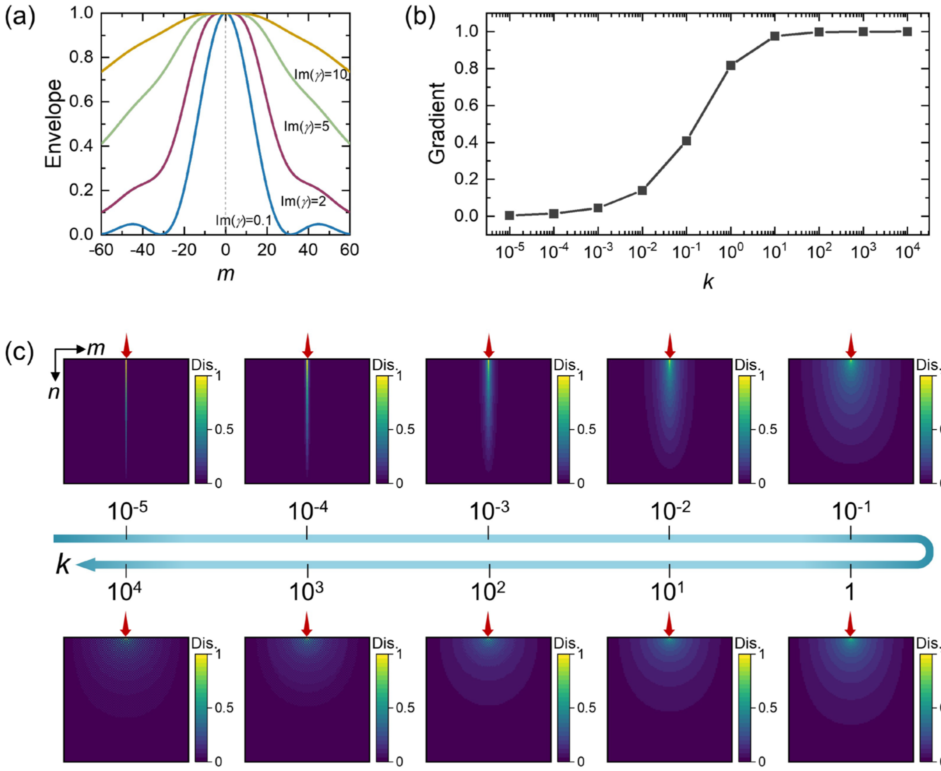

Figure S2. Localization strength of static deformation fields. (a) Wave packet envelopes with increasing $\mathrm{Im}(\gamma)$, where $\delta q = 0.1$. (b) Gradient of the real decay spectrum at $q_0 = 0$ in symmetric X-braced lattices. (c) Simulated bulk displacement fields under an applied point load for various stiffnesses.

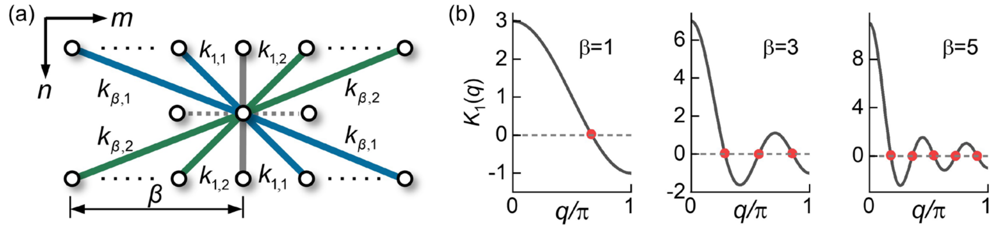

Figure S3. X-braced lattices with random coupling effects. (a) Schematic of the associate cell of a planar lattice with square grid and random set of diagonal couplings. (b) Dispersion of $K_1(q)$ in the half BZ with different degrees of non-locality, β. The associated zero points are marked by the red dots.

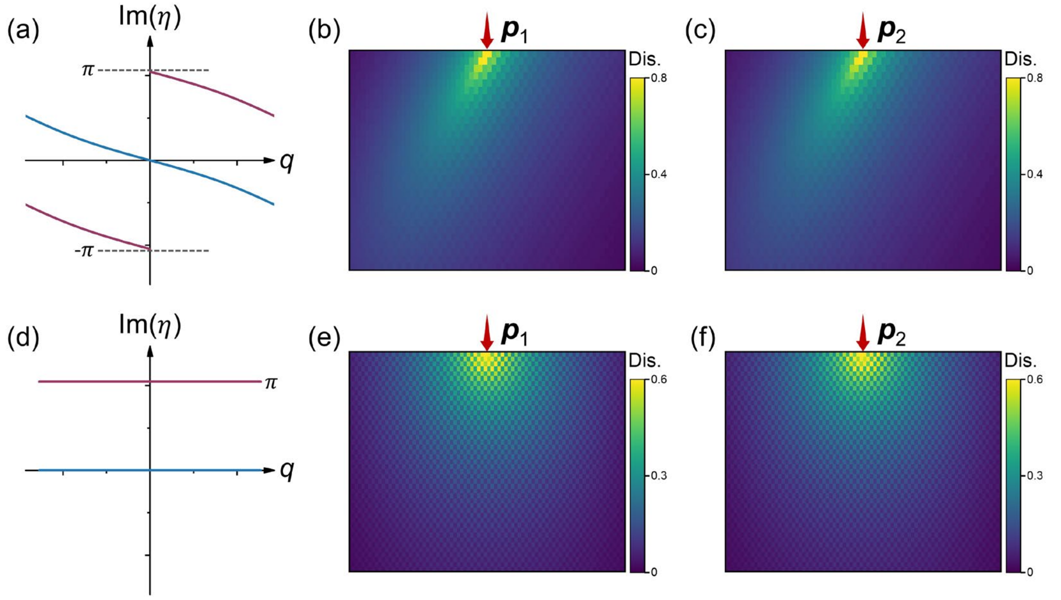

Figure S4. Wave packet transmission in diatomic rhombus lattices. (a and d) Imaginary parts of the decay spectrum. (b and e) Simulated bulk displacement fields exerted by $\boldsymbol{p}_1$. (c and f) Simulated bulk displacement fields exerted by $\boldsymbol{p}_2$.

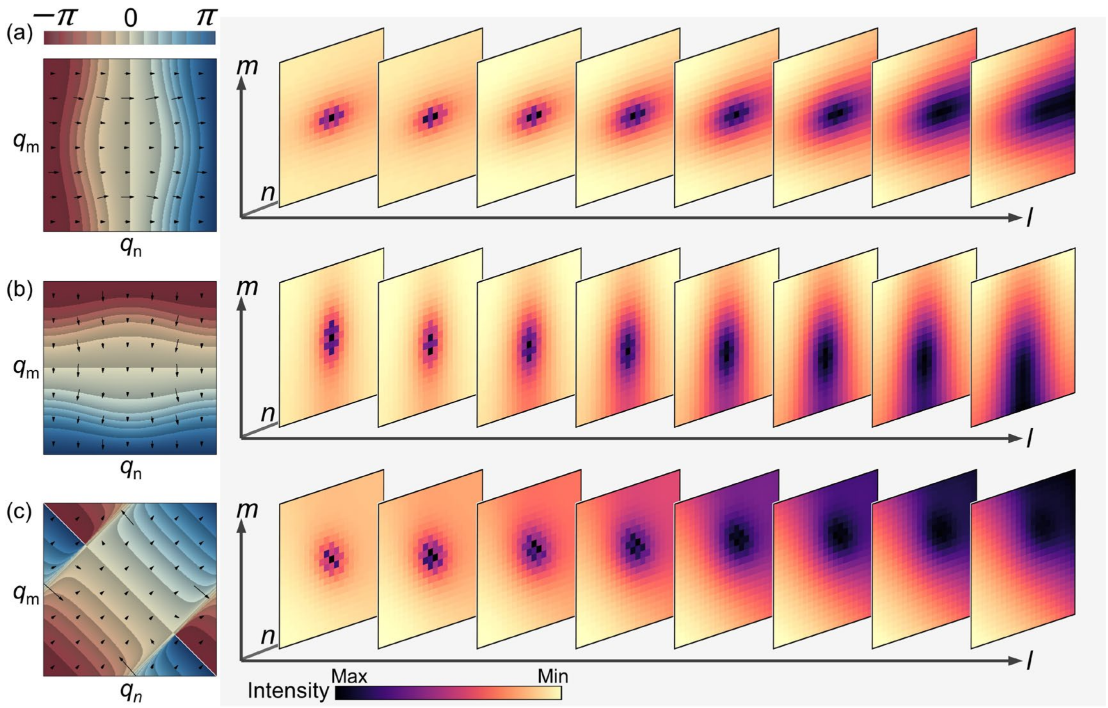

Figure S5. 3D static Rayleigh model in layered lattice materials. (a–c) Left panels depict the imaginary parts of the decay spectra in the BZ, with the gradient of the spectrum (i.e., the group velocity) marked by the black arrows. Right panels depict the real-space deformation plots under a concentrated point load applied on the center of the top layer ($l = 0$). The displacement intensities along the l axis have been renormalized for better visualization. The stiffnesses are $(k_1, k_2, k_3, k_4) = (1,1,2,6)$ for (a), $(6,2,1,1)$ for (b), and $(1,6,1,6)$ for (c), respectively.

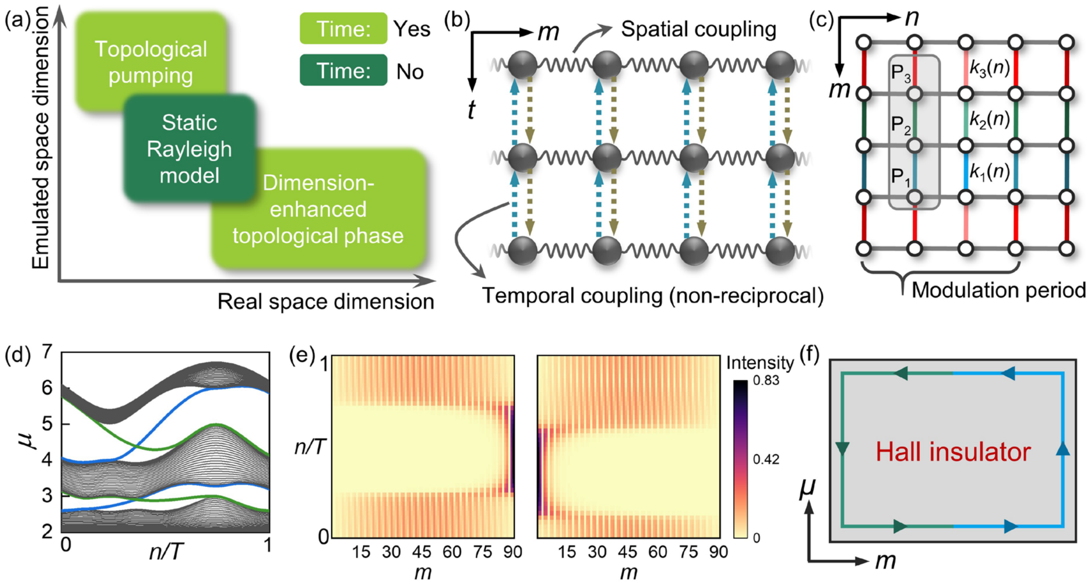

Figure S6. Topological phases in synthetic dimensions. (a) Comparison of three topological phases of matter based on their real space dimensions and the emulated space dimensions, as well as the time relevance. (b) A 1D mass-spring model with discretized time. The asymmetric couplings between the different time instants arise from the velocity-related terms like viscous damping. (c) A planar square lattice with three nodes per unit cell (gray shaded) and periodically modulated vertical stiffnesses as a function of n. (d) OBC spectrum of a finite square lattice with 30 unit cells (90 lattice sites) along the m axis, where the bulk and edge modes are marked by the gray and blue/green lines, respectively. Other parameters are $k=1$, $k_d=0.5$, $\omega=\pi/20$, $\varphi_1=0$, $\varphi_2=\pi/2$ and $\varphi_3=\pi$, respectively. (e) Adiabatic evolution of the edge states during a modulation cycle, with the period being $T=2\pi/\omega=40$. Left and right panels correspond to the blue and green lines in the top band gap of (d). (f) Schematic of a 2D Hall insulator in the synthetic space spanned by m and μ, with a chiral edge state circulating the boundaries counterclockwise. The green and blue lines correspond to the edge modes resided in the top band gap of (d).

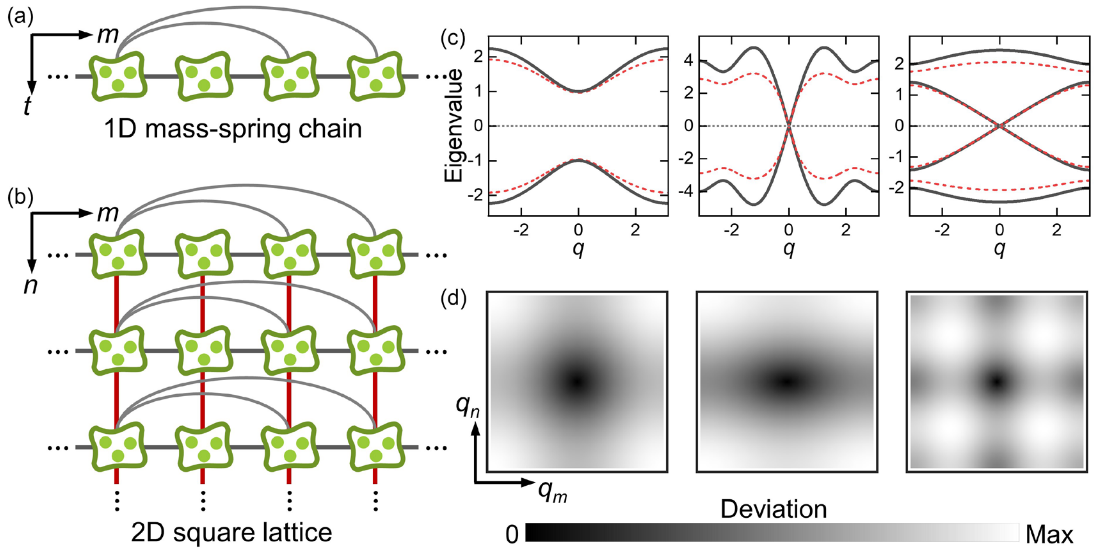

Figure S7. Low-energy physics in static Rayleigh model. (a) Schematic of a 1D mass-spring chain with arbitrary unit cell structures and coupling effects. (b) Corresponding 2D square lattice as a stack of (a) along the n axis, with the nearest interchain couplings marked by the red bars. (c) Vibration spectra ($\omega - q$, marked by the solid black lines) of a 1D monoatomic mass-spring chain with ground springs (left panel), a 1D monoatomic mass-spring chain with second- and third-nearest neighbor couplings (middle panel), and a 1D diatomic mass-spring chain (right panel). The dashed red lines denote the associated decay spectra ($\eta - q$) of the stacked square lattices. (d) Deviation between the vibration spectra of a 2D square lattice with equal stiffnesses (left panel), a 2D square lattice with larger stiffness along the vertical direction (middle panel), a 2D square lattice with second-nearest neighbor couplings (right panel), and the corresponding decay spectra of the layered 3D cubic lattices. The decay spectra coincide with the vibration spectra at the BZ center.

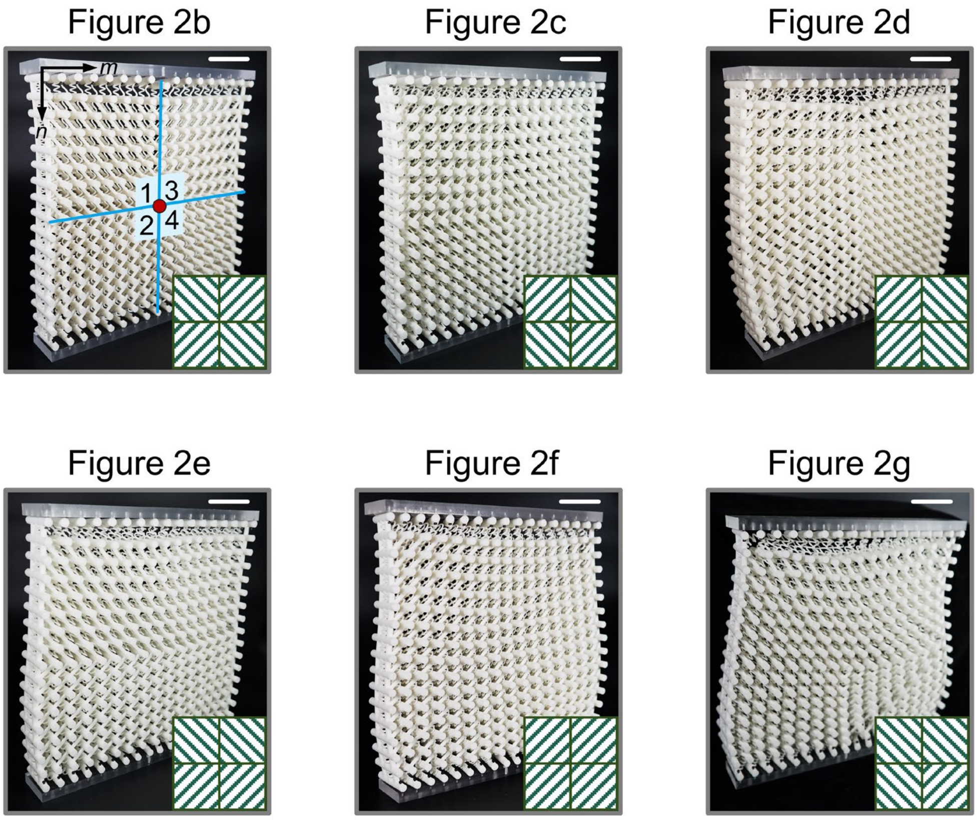

Figure S8. Photographs of the 3D-printed frame-like lattices composed of four subparts, with scale bars measuring 7cm. Insets are schematics of the direction of cylindrical beams in each subpart.

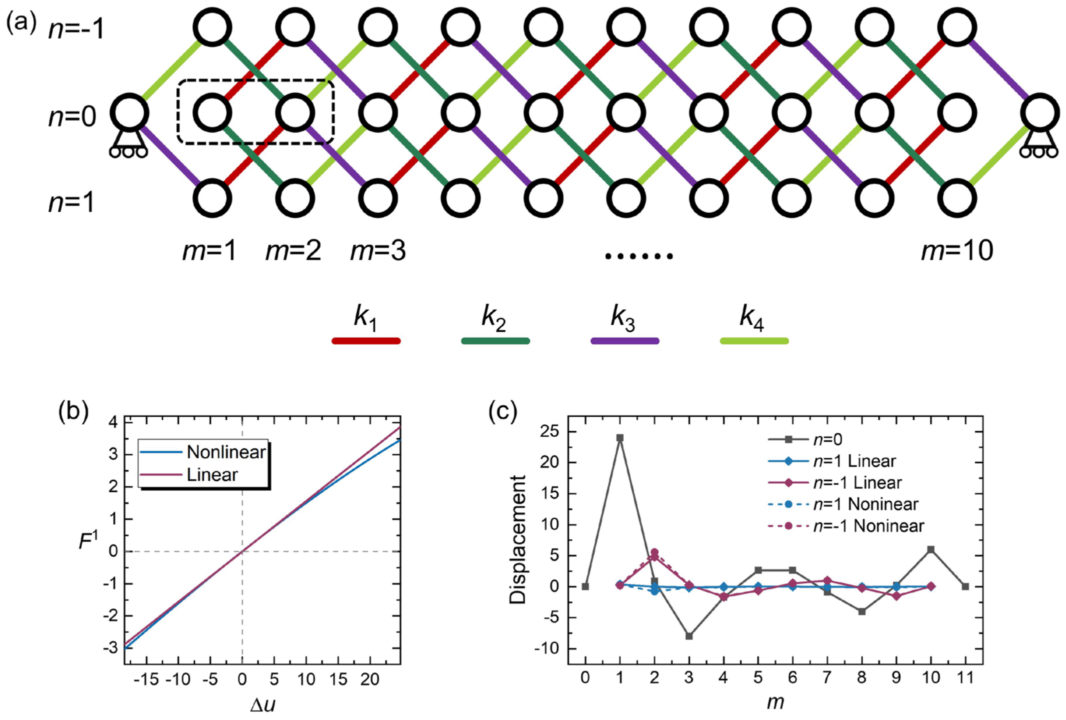

Figure S9. Nonlinear effects in the truss-like lattice. (a) Illustration of the experimental setup. The dashed box denotes the unit cell, and m is the index of nodes. (b) Linear and nonlinear force-displacement curves of bar k_1. (c) Linear and nonlinear displacement responses at $n = \pm 1$.
